# Platinum‐Doped Carbon Nitride‐Loaded Poly(*N*‐Isopropylacrylamide) Hydrogel Thin Films for Green Hydrogen Production Systems: Morphological Study for Higher Efficiency

**DOI:** 10.1002/cssc.202501550

**Published:** 2025-09-01

**Authors:** Morgan P. Le Dû, David P. Kosbahn, Thomas Baier, Julija Reitenbach, Qi Zhong, Apostolos Vagias, Robert Cubitt, Narendra Chaulagain, Karthik Shankar, Hagen Übele, Katharina Krischer, Peter Müller‐Buschbaum

**Affiliations:** ^1^ TUM School of Natural Sciences Department of Physics Chair for Functional Materials Technical University of Munich James‐Franck‐Str. 1 85748 Garching Germany; ^2^ National Base for International Science and Technology Cooperation in Textiles and Consumer‐Goods Chemistry & Zhejiang Provincial Engineering Research Center for Green and Low‐carbon Dyeing & Finishing Zhejiang Sci‐Tech University Hangzhou 310018 P. R. China; ^3^ Institut Laue‐Langevin 71 avenue des Martyrs Grenoble 38042 France; ^4^ Department of Electrical and Computer Engineering University of Alberta Edmonton AB T6G 1H9 Canada; ^5^ TUM School of Natural Sciences Department of Physics Nonequilibrium Chemical Physics Technical University of Munich James‐Franck‐Str. 1 D‐85748 Garching Germany

**Keywords:** graphitic carbon nitride, grazing incidence small‐angle neutron scattering, hydrogen evolution, photocatalysis, poly(*N*‐isopropylacrylamide), thin films, water splitting

## Abstract

Photocatalytic water splitting enables the generation of green hydrogen (H_2_). In this framework, water and sunlight are the sustainable sources. Photocatalyst‐loaded hydrogel materials have already shown their potential as a water storage and catalyst host matrix for H_2_ production. This study explores the thin film geometry of such systems to demonstrate the scalability of photocatalysis. Graphitic carbon nitride is used as a catalyst and combined with platinum as a co‐catalyst. The resulting catalytic centers are introduced in poly(*N*‐isopropylacrylamide) hydrogel thin films. First, the swelling behavior of the resulting hybrid hydrogels is studied under high relative humidity, and the influence of different catalyst loadings is discussed. Then, time‐of‐flight neutron reflectometry is used to access the vertical material composition inside the hybrid thin film in the dry state, which shows an enrichment layer of catalyst at the substrate–bulk interface. Operando grazing incidence small‐angle neutron scattering displays the microscopic changes happening under heavy water (D_2_O) vapor and light irradiation. Next, gas chromatography demonstrates the potential of the studied hydrogel films by determining their H_2_ production rates. The recorded H_2_ production is correlated to the microstructure analysis and reveals the importance of the observed catalyst enrichment layer.

## Graphical abstract

Green hydrogen production mediated by hydrogel thin films prepared from poly(*N*‐isopropylacrylamide) and graphitic carbon nitride photocatalyst with platinum cocatalyst is investigated. The swelling behavior under humid conditions for various catalyst loadings is discussed, and internal morphology is probed by neutron scattering techniques. Correlation between microstructure and hydrogen evolution indicates that a hydrophilic polymer–substrate interface enhances the hydrogen generation.

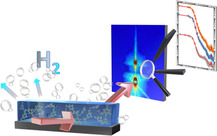

## 1 Introduction

Hydrogen (H_2_) is one of the most versatile products in terms of usage. Its significant consumption happens in the industry sector, for example, when employed in the Haber–Bosch process, hydrogen is used for ammonia production as fertilizer;^[^ ^1^, ^2^, ^3^, ^4^ ^]^ H_2_ is also a key reactant in the direct reduction of iron in the steel branch,^[^ ^1^, ^2^, ^5^, ^6^ ^]^ and it is also exploited for the production of methanol in the chemical field.^[^ ^1^, ^2^, ^7^, ^8^, ^9^ ^]^ In addition, the refining sector also widely uses hydrogen, for example, in the hydrodesulfurization process to treat natural gases, diesel, and fuel oils.^[^ ^10^, ^11^, ^12^, ^13^ ^]^ In the last decades, hydrogen has been considered as a fuel in transportation,^[^ ^1^, ^2^, ^14^, ^15^ ^]^ heating applications,^[^ ^16^, ^17^ ^]^ and electricity generation.^[^ ^1^, ^2^ ^]^ These intense usages have drastically raised the global hydrogen demand, leading to a large variety of production routes. Nowadays, production is dominated by the unabated use of nonrenewable resources such as fossil fuels or natural gases by exploiting coal and methane, leading to massive emissions of greenhouse gases. This high‐emission hydrogen is commonly called “grey hydrogen”.^[^ ^18^, ^19^, ^20^ ^]^ To overcome these significant emissions, processes are explored that allow the production of so‐called “green hydrogen” when only renewable energies are involved and no harmful side products are emitted.^[^ ^1^, ^21^ ^]^

The present study focuses on hydrogen production via photocatalysis, where visible light and water are the two required durable resources. The photocatalysis of water, also known as the water splitting reaction, is a redox process, where water molecules, H_2_O, are split into H_2_ and O_2_, that is, the reduction of water. To successfully break H_2_O molecules, an energy larger than 1.23 eV is required, representing a wavelength larger than 1.010 μm, that is, outside the visible light spectrum.^[^ ^21^, ^22^, ^23^ ^]^ Overpotential losses and charge transfer limitations typically constitute an additional energy barrier that hinders water splitting. A suitable photocatalyst generates electron‐hole pairs under visible‐to‐near infrared light with the requisite energy to satisfy the Gibbs free energy requirement for water splitting. An electrocatalyst helps to lower activation energy barriers, enabling the water‐splitting reaction to be driven closer to 1.23 V vs NHE (versus Normal Hydrogen Electrode). Photocatalysts for water splitting have some essential prerequisites in order to be scaled up to industry requirements. They should have a low electronic bandgap, a long charge carrier lifetime, be constituted of Earth‐abundant elements, be nontoxic, and be chemically and photo‐stable.^[^ ^22^, ^24^ ^]^ In this framework, this study has selected carbon nitride (C_3_N_4_, CN) as a metal‐free semiconductor, which fulfills the requirements of a photocatalyst for green H_2_ production while also displaying promising performance as an electrocatalyst.^[^ ^25^ ^]^ Carbon nitride, often called “graphitic carbon nitride” (g‐CN), consists of layers of 2D π‐conjugated polymeric structures containing tri‐s‐triazine units. The layer‐like structure is maintained by weak van der Waals forces, resulting in a graphitic geometry. In addition, the synthesis routes of CN require only Earth‐abundant‐based precursors such as urea or melamine, and the final product is obtained via thermal polycondensation. Its organic composition makes it environmentally harmless. Previous studies have already demonstrated its long lifetime under visible light exposure. In solution, g‐CN presents a bandgap of 2.7 eV, fulfilling the energy demand necessary to split H_2_O.^[^ ^26^, ^27^ ^]^ However, as a photocatalyst, g‐CN is limited by its layered structure, which hinders the charge transport in the stacking direction of nanosheets, leading to inter‐sheet photo‐carrier (electron‐hole pairs) recombination, thus decreasing the photocatalytic efficiency. Therefore, noble metals such as platinum (Pt) are introduced as cocatalysts in order to act as an electron trap and as proton reduction sites for the photocatalytic reaction. Indeed, noble metals are known to have a large work function and consequently a low Fermi level, which favors the trapping of the photogenerated electrons. Thus, Pt, with the largest work function, is the best cocatalyst candidate and acts as an electron sink. When Pt is coupled with a semiconductor, a so‐called Schottky junction is established, which leads to the alignment of the Fermi level of the semiconductor and the cocatalyst.^[^ ^28^ ^]^ The resulting junction reduces the charge recombination by extracting the electrons. In addition, the production of H_2_ is preferably happening on the Pt sites due to favorable hydrogen elimination from intermediate Pt—H species, compared to the difficult covalent elimination from a hydrogenated g‐CN surface.^[^ ^29^, ^30^, ^31^, ^32^ ^]^ For comparison, bare g‐CN without a cocatalyst exhibits a H_2_ production of less than 5 μmol g^−1^ h^−1^,^[^ ^33^ ^]^ while using Pt as a cocatalyst, the resulting system can reach a H_2_ production of up to 5000 μmol g^−1^ h^−1^.^[^ ^34^, ^35^ ^]^ However, photocatalysis of water in solution with the help of g‐CN has encountered sedimentation and/or aggregation challenges. In fact, the poor dispersibility is one of the major issues of g‐CN. The strong van der Waals interactions responsible for the π–π stacking between the sheets cause agglomeration in the liquid phase, leading to the necessity of a continuously stirred system demanding additional energy.^[^ ^36^, ^37^, ^38^ ^]^ These factors are considerable constraints to the industrial development of CN‐assisted water splitting. To overcome the mentioned drawbacks, some studies have proposed implementing hydrogel as a matrix to avoid the sedimentation problem, which also minimizes the aggregation of the catalyst particles. Such hydrogel‐supported systems have shown a hydrogel production rate reaching up to 7400 μmol g^−1^ h^−1^.^[^ ^34^, ^39^, ^40^, ^41^, ^42^, ^43^ ^]^ The observed increase in the evolution rate can therefore be associated with the improved dispersibility of the catalyst provided by the 3D scaffold of the polymer matrix. Hydrogel materials are known to be intrinsically hygroscopic, enabling a second functionality, namely water storage, which is useful for the water splitting reaction.^[^ ^44^, ^45^ ^]^ The water retentivity of hydrogels is particularly beneficial in very hot and very cold climates, where the ensuing evaporation and freezing dynamics of water, respectively, are slowed down. This study aims to explore the thin film geometry already widely used in industry and build the hydrogel film from poly(*N*‐isopropylacrylamide) (PNIPAM), an easily accessible, well‐known polymer. Indeed, the wealth of knowledge about PNIPAM has already demonstrated a good water absorption capacity as well as a high stability of PNIPAM films in the hydrated state.^[^ ^46^, ^47^, ^48^, ^49^ ^]^ The resulting studied PNIPAM hydrogel is classified as a physical hydrogel where polymer chain entanglements and hydrogen bondings maintain the polymeric scaffold; therefore, the present hydrogel is not built from any crosslinking agents. Hence, the 3D polymer network is used as a host matrix for the CN particles, and the hygroscopy of the polymer gel is employed as a water storage for the water splitting reaction. This study investigates the impact of the introduction of CN in PNIPAM hydrogel films on the water absorption behavior of the film with the use of spectral reflectance (SR), where the in situ thickness of CN‐loaded films is recorded under exposure to water vapor. Then, the vertical material distribution inside the films is determined by time‐of‐flight neutron reflectometry (ToF‐NR). In order to study the long‐term viability of CN‐PNIPAM hybrid films, the microstructure changes in operando conditions are characterized by grazing incidence small‐angle neutron scattering (GISANS). Neutron‐based characterization techniques are powerful tools that enable the resolution of sample characteristics in a nondestructive way. The large neutron beam size allows for studies on a large sample area, which brings excellent sampling statistics. In addition, the large penetration depth of neutrons allows the resolution of buried morphologies. Hence, combining ToF‐NR and GISANS gives access to information about the material arrangement inside the films^[^ ^48^, ^50^, ^51^ ^]^ and lateral distribution within the films on a nanometer scale by contrast‐induced with deuterated water (D_2_O), which is absorbed by the hydrogels.^[^ ^51^, ^52^, ^53^ ^]^ Finally, the water splitting efficiencies are obtained by gas chromatography (GC), where the produced amount of H_2_ is recorded, allowing us to determine the H_2_ evolution rate of such systems.

## 2 Results and Discussion

### 2.1 Swelling Behavior of the Hybrid Films

**Figure 1:**
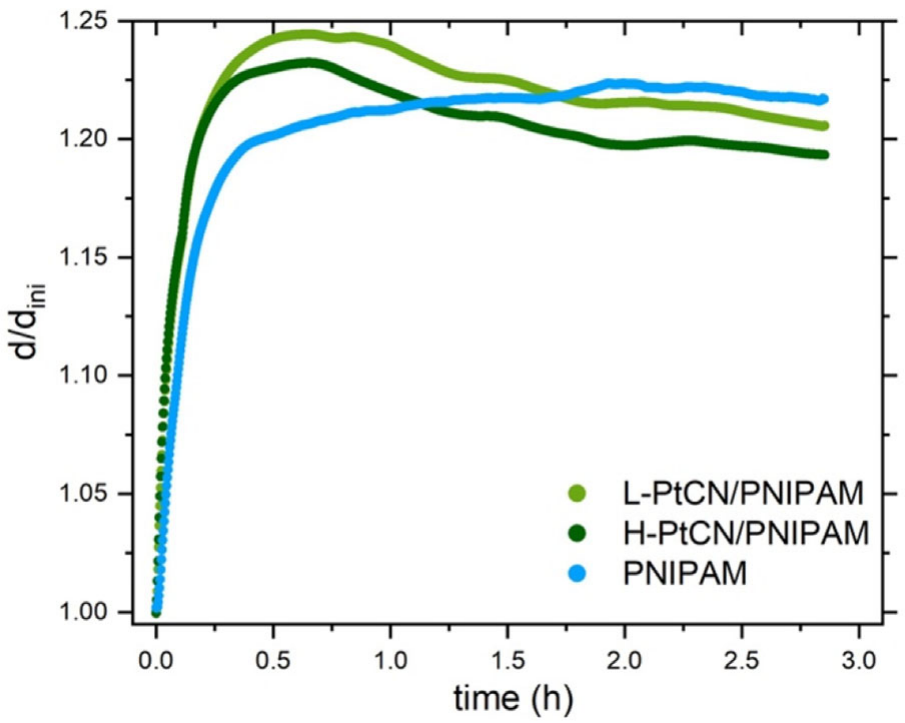
Swelling ratio evolution of L‐PtCN/PNIPAM (light green), H‐PtCN/PNIPAM (dark green), and PNIPAM (blue) film upon D_2_O vapor exposure.

Three samples are compared to study the influence of the introduction of Pt‐CN into PNIPAM films on the swelling behavior of the hydrogel films. One pure PNIPAM film is prepared and used as a reference sample, and two Pt‐CN‐loaded PNIPAM films are fabricated with two different Pt‐CN loadings, namely a PNIPAM film with a high concentration of Pt‐CN (H‐PtCN/PNIPAM) and a PNIPAM film with a low concentration of Pt‐CN (L‐PtCN/PNIPAM). Subsequently, each film is placed in a custom‐made chamber connected to a gas flow setup to enable humidity control of the sample environment.^[^ ^54^ ^]^ The whole experiment is performed at a constant temperature of ≈19 °C, maintained and monitored by a circulating water bath. All films are dried under N_2_ for 1 h before exposure to a continuous flow of 1 L min^−1^ of D_2_O vapors. The thickness evolutions are probed over time using SR with a time resolution of 10 s. **Figure** **1** shows the swelling ratio for the three samples. The swelling ratio is defined as *d(t)/d*_ini_, where *d(t)* is the film thickness measured under D_2_O vapor exposure at a given time of the experiment, and *d*_ini_ is the initial film thickness measured at the so‐called dried state when the sample has been exposed to a dry N_2_ stream for 1 h.

Figure 1 shows an immediate increase in the swelling ratio after introducing the D_2_O vapor. The films absorb the surrounding water, causing a vertical expansion, that is, an increase in film thickness. A pseudo‐equilibrium is reached after 30 min. This state appears to mark the end of the fast stretching of the polymer chains within the films due to the water incorporation and the beginning of a slower water sorption, where polymeric units rearrange in order to host more water molecules. This early‐stage maximum swelling ratio appears to be bigger in the case of the Pt‐CN‐loaded films. This observation can be attributed to the presence of carboxyl (—COOH) end groups in the catalyst particles.^[^ ^55^ ^]^ Indeed, such functional groups are known to be hydrophilic and, therefore, able to make hydrogen bonds with water, facilitating the sorption of a larger amount of water than a non‐Pt‐CN loaded PNIPAM film. In addition, the introduction of the catalyst particles may have led to the formation of nanometric pores when deposited via spin coating. These pores can be considered as tiny voids, which facilitate the absorption of water vapors. However, Figure 1 shows that this maximum swelling ratio tends to relax to a smaller value upon longer exposure times. Hence, the Pt‐CN‐loaded samples seem to undergo a relaxation where further microscopic rearrangements occur, while the pure PNIPAM film keeps swelling slowly until reaching equilibrium. These mentioned rearrangements can possibly be related to a higher polymer chain mobility in the swollen state, enabling small displacement of the catalytic particles. The different swelling behaviors observed between H‐PtCN/PNIPAM and L‐PtCN/PNIPAM films suggest that the influencing factor is the amount of Pt‐CN particles present inside the thin film. Indeed, the fact that L‐PtCN/PNIPAM reaches a higher swelling ratio than H‐PtCN/PNIPAM points toward a steric hindrance within the thickness of the samples, preventing more water molecules from being absorbed. This interpretation explains the highest water sorption capacity of the pure PNIPAM film. The Pt‐CN particles are taking up space in the polymer film, and that occupied space is therefore no longer available to host water molecules. Considering the steric factor for the Pt‐CN‐loaded samples, the observed relaxation of the swelling ratio can be attributed to a reorganization of the Pt‐CN particles aiming for the most favorable arrangement. Once fully hydrated, the Pt‐CN‐loaded films allow microscopic displacements of the catalytic centers, resulting in a decrease in the swelling ratio.

### 2.2 Inner Morphology

To further understand the observed swelling behavior of the hybrid hydrogel films, an investigation of the inner film morphology is required. Therefore, two neutron‐based characterization techniques are selected: ToF‐NR and GISANS. Combining those two methods enables a vertical and lateral study of the inner morphology of such hybrid hydrogel films. In addition, choosing neutrons as a probe prevents beam damage, often encountered when characterizing sensitive polymers with X‐rays.

#### 2.2.1 Vertical Material Distribution

**Figure 2:**
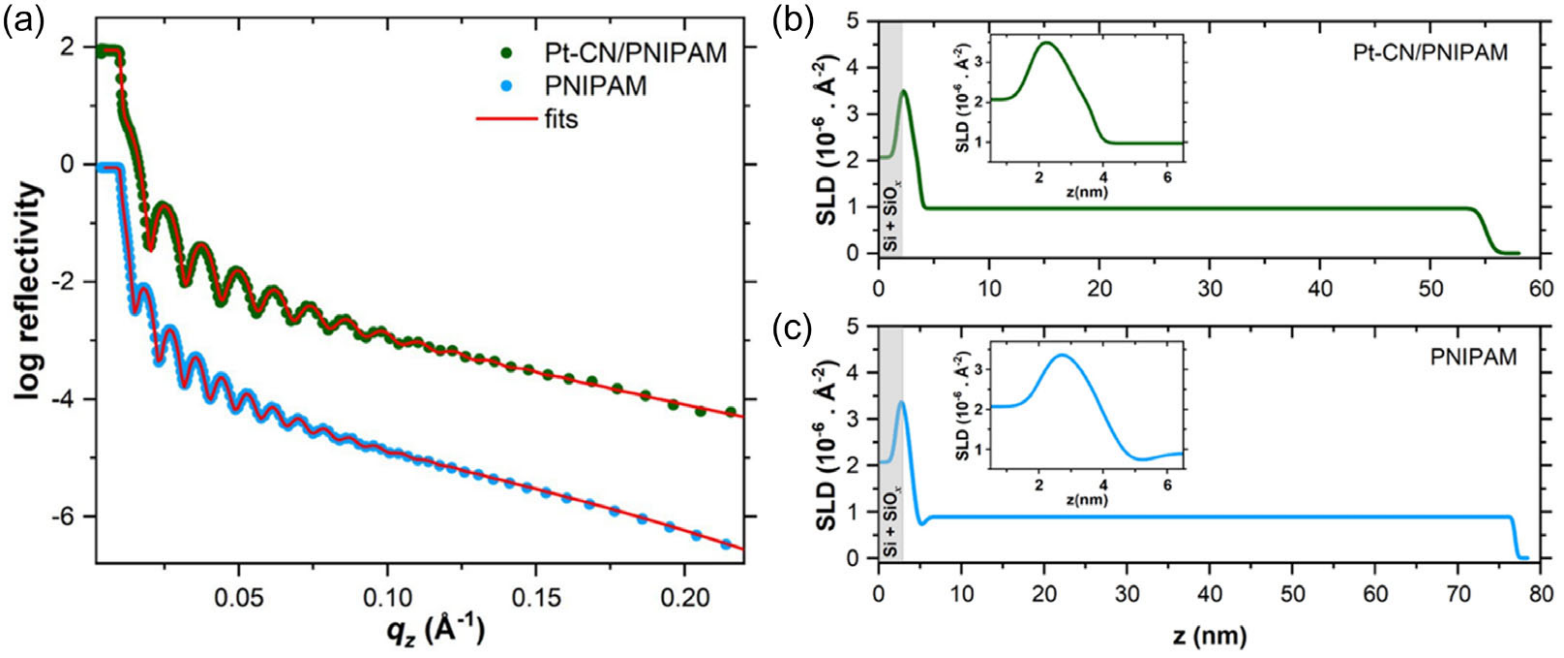
a) ToF‐NR data of PNIPAM/Pt‐CN (green dots) and PNIPAM (blue dots) with their corresponding fits (red lines); SLD profiles obtained from these fits of b) Pt‐CN/PNIPAM and c) PNIPAM thin films.

ToF‐NR is a powerful tool that allows the study of the scattering length density (SLD) distribution of thin film samples along the direction normal to the substrate. The SLD depends on the elemental composition and the density of a given material and, therefore, can be considered as its fingerprint. Furthermore, combining two neutron incident angles for the ToF‐NR measurements leads to the coverage of a broad range of momentum transfers *q*_z_, enabling a fine determination of the entire vertical film distribution. The detailed settings of the ToF‐NR measurements can be found in the Supporting Information. Once obtained, the vertical SLD profile grants detailed information about the materials arrangement within the thin film. Two thin films are compared with the neutron methods: A pure PNIPAM thin film is used as a reference, and a hybrid hydrogel thin film (Pt‐CN/PNIPAM) containing catalysts. Both samples are studied under the same conditions, namely, at a temperature of 19 °C and after exposure to a constant stream of dried N2, removing the potential absorbed water from the hygroscopic films. **Figure** **2**a shows the ToF‐NR data of PNIPAM and PNIPAM/Pt‐CN thin films in a dry state.

The well‐defined Kiessig fringes collected from both samples testify the smoothness of the thin films. A layer model is used to fit the reflectivity data. This model is composed of a superposition of layers—in detail, starting with the Si substrate and its native oxide layer, SiO_*x*_, followed by an interface layer between the substrate and the bulk part of the film, then the bulk layer of the thin film, and finally, an interface layer between the bulk part of the film and the air, often representing the smoothness of the upper surface. Figure S7, Supporting Information, illustrates the layer model used to fit the ToF‐NR data. The obtained fit parameters enable to draw the corresponding SLD profiles of the two samples, as shown in Figure 2b,c. Since both samples have the same substrates, the SLD distribution is identical near 0 nm. An interesting difference to note appears at the substrate‐bulk interface layer of Pt‐CN/PNIPAM and PNIPAM samples (see insets in Figure 2b,c). The SLD variation between the native oxide layer of the substrate and the bulk part of the film is not constant for both samples. In the case of Pt‐CN/PNIPAM, a slight shoulder appears in the SLD, corresponding to a thin layer with a higher SLD value than the bulk layer. This small SLD increase is attributed to an enrichment layer of a specific compound. Neutron‐based characterization techniques, such as ToF‐NR, grant a contrast variation where the SLD, which depends on the probed materials’ mass density and the elemental constitution, is directly measured. Hence, knowing the theoretical SLD of Pt‐CN and PNIPAM enables us to attribute the SLD variations to different layers and interfaces. Because the SLD value of the enrichment layer (2.17 × 10^−6^ Å^−2^) is higher than the SLD of PNIPAM (0.81 × 10^−6^ Å^−2^) and since Pt‐CN has the highest SLD of the introduced materials, this layer can be seen as a thin enrichment of the catalytic centers at this specific interface, while the SLD profile at the substrate‐bulk interface of PNIPAM samples shown in Figure 2c exhibits a different trend. There appears to be a decrease in the SLD for this layer, thus corresponding to a material with a lower SLD than the polymer bulk layer. This behavior is characteristic of an H_2_O enrichment layer at this interface.^[^ ^49^ ^]^ Indeed, it is well known that hydrogels such as PNIPAM are hygroscopic, collect water from the surroundings, and store it at a highly hydrophilic interface between the SiO_*x*_ and the bulk polymer layer.^[^ ^49^ ^]^ Hence, the negative H_2_O SLD of −0.561 × 10^−6^ Å^−2^ is causing the observed SLD reduction. It is reasonable to estimate that the enrichment layer in Pt‐CN/PNIPAM also contains a small quantity of water, as the hygroscopy of the hydrogel and the hydrophilic substrate surface are conserved. The SLD of this substrate‐bulk interface, the PNIPAM sample, is taken as a reference to evaluate the amount of Pt‐CN in this enrichment layer. The thickness difference between these two films is considered to have a negligible impact on the resulting uptaken H_2_O of this interface. The quantity of catalyst at this interface is determined from its volume fraction expressed as $\Phi_{\text{Pt‐CN}}=\frac{SLD_{\text{meas}}-SLD_{\text{PNIPAMlayer}}}{SLD_{\text{Pt‐CN}}-SLD_{\text{PNIPAMlayer}}}\times 100$, where *SLD*_meas_ is the SLD found from the fit parameters, *SLD*_Pt‐CN_ is the calculated SLD of the photocatalyst, and *SLD*_PNIPAMlaye*r*_ is the SLD obtained from the fit of the H_2_O enrichment layer found in the PNIPAM sample. As a result, the substrate‐bulk interface is occupied by a volume fraction of Pt‐CN of Φ_Pt‐CN_ = 32.8%. Concerning the bulk layer, the SLD contribution observed for the PNIPAM sample in Figure 2c agrees with the calculated SLD of PNIPAM homopolymer. The fit gives a value of 0.82 × 10^−6^ Å^−2^ for the PNIPAM bulk layer, whereas a value of 1.16 × 10^−6^ Å^−2^ is found for the bulk layer of Pt‐CN/PNIPAM thin film. The SLD contributions of a binary mixture can be treated in order to determine the volume fraction of a given compound in the mixture. The volume fraction of Pt‐CN within the polymer thin film bulk layer is calculated as follows: $\Phi_{\text{Pt‐CN}}=\frac{SLD_{\text{meas}}-SLD_{\text{poly}}}{SLD_{\text{Pt‐CN}}-SLD_{\text{poly}}}\times 100$, *SLD*_poly_ is the calculated SLD of PNIPAM homopolymer. According to the latter equation, the volume fraction $\Phi_{\text{Pt‐CN}}$ in the bulk layer of the loaded film is 5.4%. The aggregation tendency and the hydrophilicity of Pt‐CN can explain the concentration gradient of catalysts within the entire film. As already mentioned, this compound has —COOH end groups,^[^ ^55^ ^]^ which preferentially make hydrogen bonds with water molecules. Hence, the catalytic centers appear to be located where the water is the most abundant. The upper surface of the film is affected by the introduction of the catalyst centers. In fact, the steep decrease in the SLD profile at the polymer‐air interface observed in the PNIPAM sample demonstrates the high smoothness of the thin film, while in Figure 2b the considered slope has a moderate steepness for the hybrid hydrogel film, attesting a roughening of the upper layer of the sample, which is attributed to the presence of catalyst particles in the thin film.

#### 2.2.2 Lateral Microstructure

GISANS contributes to the second step of the inner morphology investigation. It enables the resolution of characteristic nanostructured objects in terms of their sizes and lateral arrangements within thin films with good statistics. This characterization focuses on the two loaded samples, H‐PtCN/PNIPAM and L‐PtCN/PNIPAM, already presented during the swelling experiment with SR, where the dry, D_2_O hydrated, and light irradiated states are studied. Figure S8, Supporting Information, shows the 2D GISANS data recorded for each state of both samples. Scattering contrast between the polymer matrix and the catalytic centers allows to determine the inner film morphology from horizontal line cuts of the 2D GISANS data taken at the Yoneda region.^[^ ^53^ ^]^

Deuterated solvents such as D_2_O grant a contrast enhancement in GISANS, allowing for the detection of fine refinements of the morphologies in the hydrated state. The fast swelling of the samples resulting from the D_2_O vapor exposure does not permit long data acquisition times. Hence, 2 h of the hydration kinetics are followed with a time resolution of 10 min, whereas the irradiation process is probed for 1 h with a time resolution of 10 min. The kinetic processes between those states are determined from vertical line cuts of the 2D GISANS data taken at a fixed *q*_y_ (*q*_y_ = 0 nm^−1^), where the position of the Yoneda peak is tracked. Exemplary 2D GISANS data are shown in Figure S10, Supporting Information, where the areas of interest for the data treatment are highlighted. Finally, the samples are exposed to UV irradiation to trigger the photocatalysis process. The light irradiation is provided by a 360 nm UV LED mounted in the custom‐built setup. In order not to break the high humidity inside the sample environment, the D_2_O vapor stream is kept constant.

**Figure 3:**
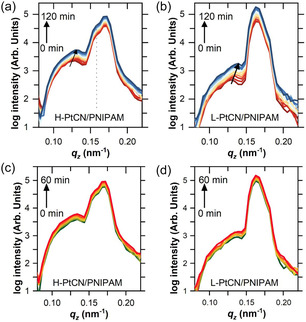
Vertical line cuts of the 2D GISANS data probing the kinetic processes of a,c) H‐PtCN/PNIPAM and b,d) L‐PtCN/PNIPAM thin films during (a,b) D_2_O vapor exposure and (c,d) UV irradiation.

**Figure 4:**
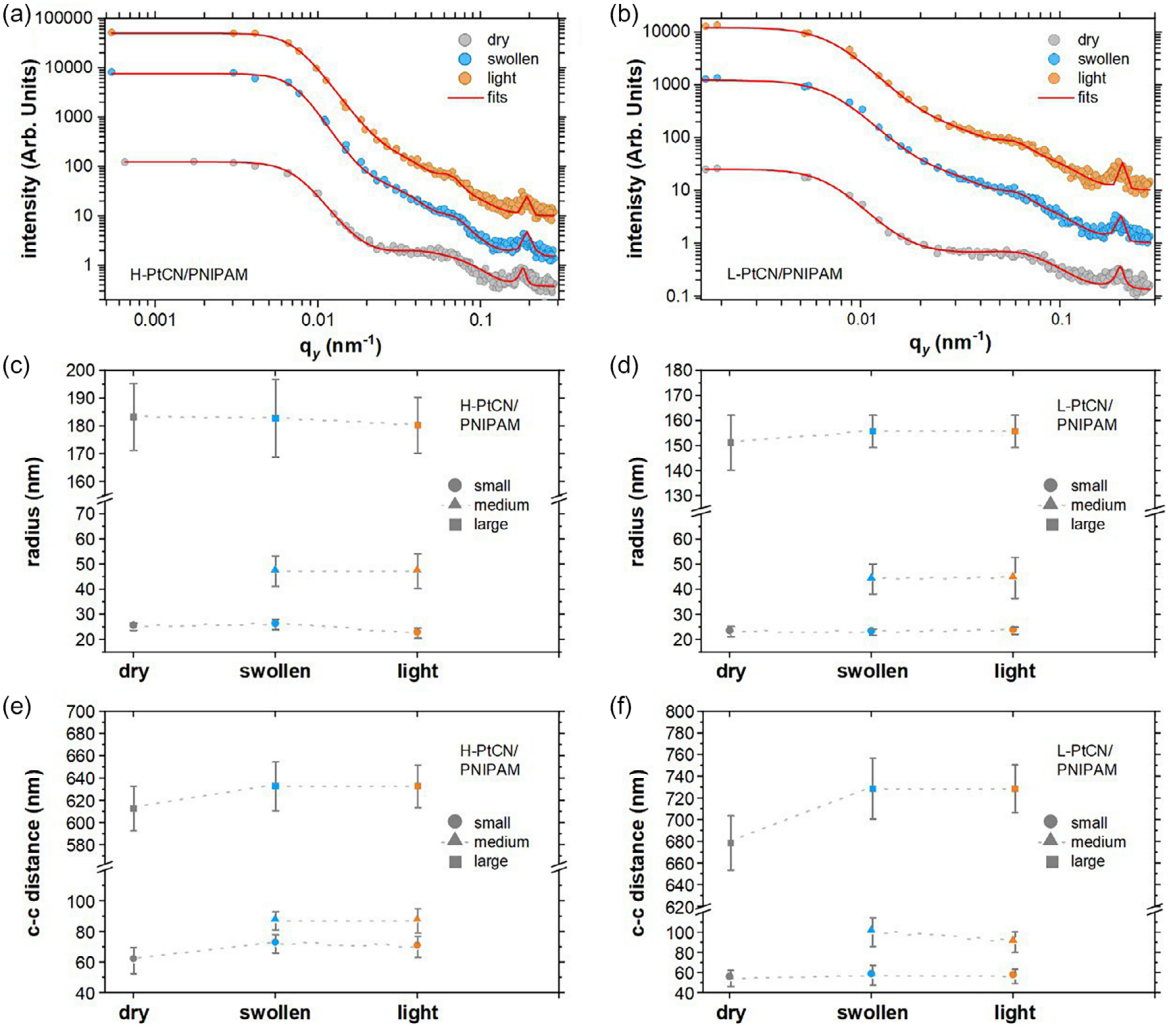
a,b) Horizontal line cuts of the 2D GISANS data at the Yoneda position of H‐PtCN/PNIPAM and L‐PtCN/PNIPAM films for each state of the characterization: dry (gray), swollen (hydrated, blue), and light irradiated (yellow) state. The red lines represent the associated fits. c,d) Cylinder radii extracted from the fits for the assumed objects and e,f) respective center‐to‐center (c‐c) distances.

The kinetics during light irradiation are also studied from the vertical line cuts at a fixed *q*_y_ (*q*_y_ = 0 nm^−1^). The final light‐irradiated state of the samples is statically probed as described earlier in order to obtain good statistics to proceed to horizontal line cuts on the 2D data. **Figure** **3** shows the intensity distribution along *q*_z_ for both samples during the two kinetic processes (D_2_O hydration and light irradiation). The Yoneda peak is visible in Figure 3 at *q*_z_ = 0.126 and 0.130 nm^−1^ for H‐PtCN/PNIPAM and L‐PtCN/PNIPAM samples, respectively. From this observation, it appears that the L‐PtCN/PNIPAM sample has a bigger loading of Pt‐CN. However, the H‐PtCN/PNIPAM sample shows a shoulder in the intensity profile close to the specular peak at *q*_z_ = 0.158 nm^−1^, which originates from a distinct layer of a high SLD component. Such a layer can be an enrichment layer of Pt‐CN, as already observed by ToF‐NR. However, for the H‐PtCN/PNIPAM sample, the Pt‐CN enrichment layer from GISANS has a higher SLD (4.97 × 10^−6^ Å^−2^) than the one observed with ToF‐NR. We attribute such deviation to the differences in the amount of catalyst introduced in the sample. Indeed, GISANS requires thicker films to enhance the scattering and to reduce the measurement time compared with ToF‐NR. Thus, the catalyst loading is higher in the case of the GISANS samples. Previous ToF‐NR studies on polyacrylamide thin films have shown that the substrate interface effect is independent of the polymer film thickness.^[^ ^49^, ^56^, ^57^, ^58^ ^]^ Consequently, the observed difference is correlated to the larger amount of catalyst in the H‐PtCN/PNIPAM sample. In addition, the L‐PtCN/PNIPAM sample does not exhibit this intensity shoulder, which supports that the Pt‐CN enrichment at the substrate–bulk layer interface is connected to the quantity of catalyst introduced in the sample. The fact that the L‐PtCN/PNIPAM film does not display this distinct shoulder suggests that the Pt‐CN‐rich layer in this sample is not thick enough to be visible under grazing incidence small‐angle geometry. Regarding the D_2_O vapor exposure, the Yoneda peak shifts toward higher *q*_z_ values, as indicated by the arrows in the plots. This shift originates from the sorption of D_2_O by the samples. Since D_2_O has a high neutron SLD, an uptake of D_2_O causes a shift of the Yoneda peak to higher *q*_z_ values. When focusing on the comparison of the Yoneda peak shift between the two samples, it appears that the L‐PtCN/PNIPAM Yoneda peak undergoes a stronger shift (Δ*q*_z_ = 0.01 nm^−1^) than H‐PtCN/PNIPAM Yoneda peak (Δ*q*_z_ = 0.007 nm^−1^), suggesting that L‐PtCN/PNIPAM absorbs more D_2_O. This observation correlates with the SR measurement. The study of the UV light exposure (Figure 3c,d) does not reveal any shifts of the Yoneda peak of both samples, meaning that the overall SLD remains constant during the process. This observation indicates that the amount of D_2_O stays constant, which is expected since the flow of D_2_O vapor is still running during the irradiation time. Excluding any expulsion of D_2_O from drying, only a change in the mass density caused by possible degradations would influence the Yoneda peak position. Therefore, neither sample appears to undergo photodegradation during the UV light exposure. Investigating the lateral morphologies within the samples is required to precisely understand the influence of hydration and light irradiation. **Figure** **4** shows horizontal line cuts of the 2D GISANS data taken at the Yoneda peak. The obtained intensity distributions are fitted with a cylindrical form factor since no other form factors led to matching fits. In addition, the Pt‐CN sheets can be pictured as stacked disks where the height of the considered cylinder is defined by the number of sheets stacked on top of each other. The detailed model used for the fits is explained in the Supporting Information. The horizontal line cuts in Figure 4a,b show identical peaks at high *q*_y_ values (*q*_y_ = 0.183 nm^−1^), independent of the sample state (dry, swollen, or illuminated). Such peaks are attributed to the transmitted intensity as seen in Figure S10, Supporting Information, which has a “cross‐shape” pattern known to originate from internal defects of the used Si substrates. Thus, these peaks are not included in the model fits of the horizontal line cuts but are considered with Gaussian peaks. Concerning the radii extracted from the fits, shown in Figure 4c,d, the dry state of both samples exhibits two cylinder sizes, namely, small and large ones. The small cylinders have a radius of 26 ± 2 and 24 ± 1 nm for H‐PtCN/PNIPAM and L‐PtCN/PNIPAM, respectively, and are attributed to small particles of CN. The large cylinders have a radius of 183 ± 12 and 151 ± 11 nm for H‐PtCN/PNIPAM and L‐PtCN/PNIPAM, respectively, and appear to be large clusters of CN.

To assess the abundance of the large clusters, the domain size distribution is plotted (Figure S11, Supporting Information). As noted from the logarithmic intensity scale, one can see that those clusters are present in a relatively low amount in comparison to the small ones. Interestingly, the cluster size is bigger in the H‐PtCN/PNIPAM sample case. Therefore, it appears that the presence of those clusters and their associated sizes are related to the amount of catalyst. The center‐to‐center distances of these objects in the dry state are shown in Figure 4e,f and reveal that the Pt‐CN catalytic centers are not aggregated since their spacing is at least twice bigger than their radius. In the swollen state, when the samples have been exposed to D_2_O vapor, the fits require the addition of one more structure to be able to match the collected data. These new cylinders have a radius between the small and large ones and, therefore, are called medium‐sized cylinders, with a size of 47 ± 6 and 45 ± 6 nm for H‐PtCN/PNIPAM and L‐PtCN/PNIPAM, respectively. It is important to note that this medium‐sized cylinder radius is about twice the size of the small cylinders. One possible explanation is an aggregation of the small‐sized Pt‐CN particles enabled by the enhancement of the polymer chain mobility in the hydrated state. Concerning the small and large cylinders, their sizes remain constant in the swollen state, whereas the associated spacings seem to be affected by the introduction of D_2_O within the samples. The large clusters appear to be more affected, especially in the case of the L‐PtCN/PNIPAM sample, where a 50 nm increase is observed from the dry to the swollen state. The spacing increase agrees with the enhanced mobility hypothesis arising from the D_2_O sorption. Furthermore, the mobility within the samples seems to be linked to the amount of Pt‐CN in the sample since the distances in the H‐PtCN/PNIPAM sample do not exhibit such a strong change from dry to swollen state. Consequently, a large amount of Pt‐CN sterically hinders the motion of the clusters and, therefore, prevents microscopic rearrangement within the samples, resulting in a lower D_2_O storage capacity, as seen with the SR characterization. The parameters extracted from the fits to the horizontal line cuts obtained from the light‐irradiated state do not display big changes. Indeed, the cylinder radii, as well as their associated spacings, are comparable to those extracted from the swollen state. Accordingly, a degradation under light irradiation is also not found for the lateral structures inside the Pt‐CN‐loaded films during probed exposure times.

### 2.3 Photocatalytic Water Splitting

**Figure 5:**
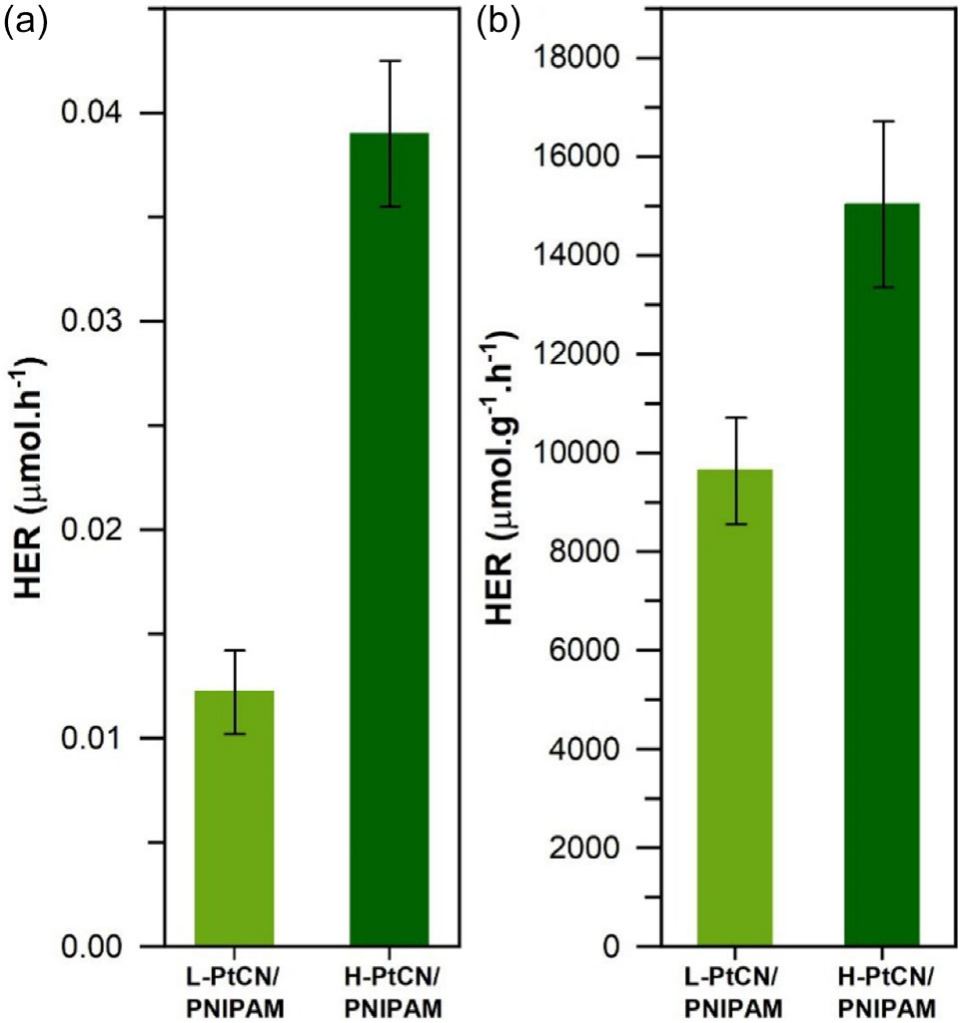
a) Absolute HER and b) HER normalized to the catalyst mass in the samples.

The H_2_ evolution of H‐PtCN/PNIPAM and L‐PtCN/PNIPAM samples is probed in order to investigate if the thin film geometry influences the H_2_ production. A custom‐built cell enabling gas detection from thin film samples is used for the photocatalytic tests, and a detailed description of the setup can be found in the Supporting Information. The gas characterization is operated by a gas chromatograph working with Argon (Ar) as carrier gas. To enable accurate detection, a sufficient amount of H_2_ needs to be produced; hence, the gas flow used for the previous characterization is not selected. Indeed, it would provide a continuous gas stream to the gas chromatograph, carrying only a small amount of H_2_. Therefore, the cell is equipped with a channel formerly filled with H_2_O. In this setting, the cell is filled with Ar gas to install the required atmosphere for the gas to be injected into the gas chromatograph. Once Ar‐filled, the cell is sealed and kept airtight, while a high relative humidity environment is built from the diffusion of H_2_O occupying the channels. Considering the slow natural diffusion of H_2_O in the cell, the swelling time is set to 24 h to ensure that the hydrated state of the sample has reached an equilibrium. Then, the light irradiation is set to 4 h. Finally, the gas from the sample environment is injected into the gas chromatograph to analyze the gas composition. The resulting H_2_ evolution rate of 0.01 and 0.03 μmol h^−1^ is measured for L‐PtCN/PNIPAM and H‐PtCN/PNIPAM, respectively (**Figure** **5**a). Such small values are expected since the exact mass of the catalyst introduced has not yet been considered. Indeed, the initial Pt‐CN solution with a concentration of 5 mg mL^−1^ is first centrifuged, where only the supernatant phase is kept. The resulting solution is filtered, and finally, only a small amount of the catalyst is deposited on the substrates. Therefore, the final concentration of Pt‐CN is determined from a calibration curve built with UV‐vis‐light spectroscopy using several solutions of known concentration, which can be found in the Supporting Information. Additionally, the material losses induced by the spin coating technique need to be considered. Hence, the amount of catalyst within the thin film samples after film deposition is estimated from the Pt‐CN:PNIPAM ratio and the dimensions of the samples; the detailed steps of the mass estimation are found in the Supporting Information.

The final H_2_ evolution rate (HER) reaches 9600 ± 1000 and 15 000 ± 1600 μmol g^−1^ h^−1^ for L‐PtCN/PNIPAM and H‐PtCN/PNIPAM (Figure 5b), respectively. Normalizing the HER by the catalyst mass enables the withdrawal of the amount of catalyst factor and focuses only on the influence of the inner morphologies observed in the samples. Indeed, one can expect a similar HER after normalization; however, H‐PtCN/PNIPAM shows the best H_2_ production rate. Thus, this difference lies in the buried morphology of the samples. The main difference that could explain these HER is the catalyst enrichment layer observed in ToF‐NR in the case of the Pt‐CN loaded sample. This observation is further confirmed by GISANS with the distinct shoulder observed in Figure 3a,c for H‐PtCN/PNIPAM, confirming the presence of this catalyst‐rich layer and suggesting a thicker enrichment layer in H‐PtCN/PNIPAM. Indeed, L‐PtCN/PNIPAM sample does not display this shoulder (Figure 3b,d) and therefore suggests a thinner catalyst‐rich not detectable in GISANS geometry. In addition, the SLD distribution within a catalyst‐loaded PNIPAM film (Figure 2b) demonstrates that this enrichment layer at the substrate polymer interface also contains water. Hence, the enrichment layer of Pt‐CN in H‐PtCN/PNIPAM observed at the substrate polymer interface is placed where the water is preferentially located in a PNIPAM thin film. The origin of this layer can be explained by the substrate cleaning procedure. In fact, by using the acid cleaning treatment plus the O_2_ plasma exposure, the surface of the resulting Si substrates becomes significantly more hydrophilic due to the presence of a thin SiO_*x*_ layer. The oxidized substrate surface forms polar silanol groups (‐Si‐OH) via hydrogen bonding with the residual water in the polymer matrix or with the carboxyl end groups of the catalyst. Therefore, the mentioned layer contains more water and more photocatalyst particles in comparison to the bulk film. Consequently, this Pt‐CN/H_2_O enrichment provides easy access to a larger amount of catalyst particles to the water, leading to a more efficient splitting of H_2_O, producing H_2_. The observed HER independent of the catalyst mass (μmol·h^−1^) is relatively low;^[^ ^34^, ^59^ ^]^ however, the dimensions of the samples are also drastically reduced. Indeed, the studied samples have a volume of the order of 0.2 mm^3^. As discussed earlier, the HER is improved when adding more catalyst to the hydrogel thin films, but it also involves a decrease in the swelling capacity of the hydrogel. Hence, the H_2_ production efficiency of the hydrogel thin film system is limited by the balance between the amount of catalyst and the water storage capacity of the films. When focusing on the HER with the catalyst mass consideration (μmol·g^−1^ h^−1^), the observed production rate is higher than the ones reported in earlier works.^[^ ^34^, ^35^, ^59^ ^]^ Such differences can be explained by the peculiar design of the used CN. As seen in Figure S2d, Supporting Information, the catalyst has an enhanced absorption toward the visible‐light range. In addition, the presence of the —COOH end groups on the Pt‐CN catalyst improves its hydrophilicity and, therefore, its accessibility to H_2_O molecules for better water splitting reaction.

## 3 Conclusion

The presented study explores the potential of hydrogel thin films for photocatalytic green H_2_ production. The hydrogel film is used as a host matrix for the photocatalyst and as a water storage medium for the water‐splitting reaction. The wealth of knowledge about PNIPAM hydrogel films and carbon nitride catalysts facilitates understanding of the ongoing processes happening in the thin film during photocatalysis from the thin film geometry. The in situ SR characterization under high humidity conditions reveals that the presence of catalysts limits the water sorption of the PNIPAM hydrogel film and shows a maximum in the swelling ratio, which tends to relax toward a smaller swelling ratio at the end of the process. Such observation can be attributed to microscopic rearrangements within the films. ToF‐NR is used to assess buried interfaces inside a catalyst‐loaded hydrogel film. The SLD distribution of the loaded sample demonstrates an enrichment layer of water and catalysts at the substrate polymer interface and a small volume fraction of Pt‐CN within the bulk part of the polymer layer. Operando GISANS confirms the presence of this distinct enrichment layer of the catalyst in the H‐PtCN/PNIPAM sample and the smaller swelling of this sample. In addition, GISANS reveals an aggregation of the small‐sized catalytic centers in the hydrated state and stable inner morphologies under light irradiation. Finally, a higher H_2_ production rate is found for the H‐PtCN/PNIPAM sample, as shown with GC measurements. This observation is described by the presence of the enrichment layer in H‐PtCN/PNIPAM and correlated to an easy access to water of a larger amount of Pt‐CN particles.

## 4 Experimental Section

4.1

4.1.1

##### Materials and Sample Preparation

*Materials*: Silicon wafers (p/Bor, <100>, *d* = 525 ± 25 μm, 10–20 Ω × cm) from Si‐Mat, Kaufering, Germany, were used as substrates for the preparation of films for spectral reflectance (SR), ToF‐NR, and GISANS characterizations. Glass slides (70 × 90 mm^2^, 1.0 mm thick, 100% OT) from Thermo Scientific, Germany, were used as substrates for the GC measurement. The substrates were precut with an area of 20 × 20 mm^2^ for SR measurements; a precut area of 70 × 70 mm^2^ was used for the GISANS and ToF‐NR characterization, and 30 × 30 mm^2^ for the GC measurement. The cleaning of the substrates was done through an acid cleaning procedure with deionized water, sulfuric acid (H_2_SO_4_, 95–98%, Aldrich), and hydrogen peroxide (H_2_O_2_, 30% aq., Roth), which were used as received. All the water used for the SR characterization was deionized to a resistivity of 18.2 MΩ × cm using a Milli‐Q Plus purification system (Merck Millipore, Burlington, USA). The PNIPAM solutions were prepared from poly‐(*N*‐isopropylacrylamide) from Aldrich (PNIPAM, M_n_ ≈ 40 000 g mol^−1^, *T*_mp_ = 96 °C) stored in the dark at 4 °C. 1,4‐Dioxane (≥99.5%, Aldrich) was used as received as a solvent for the polymer solutions. For each measurement, the polymer solutions were filtered by using hydrophobic polytetrafluoroethylene (PTFE) membrane filters with 0.45 μm pore size (Merck Millipore, Burlington, USA), while the catalyst solutions were filtered with hydrophilic PTFE membrane filters with a pore size 0.45 and 0.2 μm; the details of the sample preparations are described in the next section. Pt‐doped carbon nitride was used as a photocatalyst, and its synthesis and characterization are provided in the Supporting Information with the associated Figure S1, S2, and S3, Supporting Information. In the case of vapor exposure during GISANS and ToF‐NR characterization, D_2_O (99.95%, Deutero GmbH) was used.

*Thin film preparation*: 40 mg mL^−1^ solution of PNIPAM was prepared with 1,4‐dioxane and left on a lab shaker for 24 h to ensure homogenized dissolution of the polymer. Next, the solutions were filtered by using hydrophobic PTFE filters to remove impurities. A part of this solution is kept for the fabrication of the pure PNIPAM film, while another part is used to prepare the catalyst‐loaded films. The hybrid hydrogel films were prepared as follows: 5 mg of Pt‐CN was dispersed in 1 mL of deionized water (DI H_2_O) and sonicated for 30 min at room temperature. Once fully dispersed, the Pt‐CN solution was centrifuged for 10 min at 18 000 rotation per minute (rpm). Then, the supernatant phase of the obtained product was extracted and filtered with a PTFE hydrophilic membrane filter with a 0.2 μm pore size (Merck Millipore, Burlington, USA) to remove the possible larger particles or aggregates that could remain after the centrifugation process. Two hybrid hydrogel films for the GISANS characterizations were prepared with two different concentrations of Pt‐CN, namely H‐PtCN/PNIPAM and L‐PtCN/PNIPAM, for a high concentration of Pt‐CN in PNIPAM solution and a low concentration of Pt‐CN in PNIPAM solution. In detail, the H‐PtCN/PNIPAM sample was prepared by introducing 300 μL of the filtered Pt‐CN solution into 3 mL of the previously prepared PNIPAM solution. In comparison, 150 μL of Pt‐CN plus 150 μL of DI H_2_O were introduced into the PNIPAM solution for the L‐PtCN/PNIPAM sample. Introducing additional DI H_2_O enables an equal PNIPAM concentration in both solutions and a similar quantity of H_2_O for the hybrid hydrogel films. The resulting Pt‐CN mass introduced into the samples is obtained from a calibration curve built with UV‐vis‐light spectroscopy as detailed in the Supporting Information. The sample used for ToF‐NR was prepared differently to obtain thinner films. In brief, two samples were prepared: one PNIPAM thin film as a control sample and one Pt‐CN/PNIPAM sample. The Pt‐CN catalyst powder was first introduced in DI H_2_O (5.9 mg mL^−1^); the solution was sonicated for 1 h, then centrifuged for 10 min at 10 000 rpm, which was followed by a filtration step with a PTFE hydrophilic membrane with a pore size of 0.45 μm. To ensure a smooth film, crucial in reflectivity characterizations, another centrifugation at 18 000 rpm for 10 min and filtration with PTFE 0.2 μm pore size was performed on the Pt‐CN solution. The PNIPAM film was prepared from a 15 mg/mL polymer solution and spin coated. Pt‐CN/PMIPAM film was prepared from 3 mL of the 15 mg mL^−1^ PNIPAM solution with 400 μL of the prepared Pt‐CN solution and spin coated. For all the samples, precut silicon wafers and glass substrates were submerged in an acid bath (54 mL deionized water, 84 mL H_2_O_2_, 198 mL H_2_SO_4_) at 80 °C for 30 min, carefully rinsed with DI water, dried using N_2_, and treated with oxygen plasma (240 W) at 0.4 mbar for 20 min. Immediately afterward, thin PNIPAM film and hybrid hydrogel films were deposited via spin casting (2500 rpm, 60 s, *V* = 0.11 mL for SR samples, *V* = 3.0 mL for GISANS and ToF‐NR samples, *V* = 1.0 mL for GC samples). The thin film samples coated on glass substrates are used for the GC characterization since the used setup permits only light irradiation from the bottom; hence, the substrate needs to transmit light. The obtained thin film samples were stored in a desiccator. Figure S4, Supporting Information, shows a schematic view of the sample preparation steps.

##### Methods

*SR measurements*: SR measurements were performed using a custom‐made sample environment described in the Supporting Information, a Filmetrics F20 Thin‐Film Analyzer (KLA, Milpitas, USA) with a halogen lamp light source (*λ* = 380–1100 nm) and a spectrometer. The measurement details can be found in the Supporting Information.

*ToF‐NR and GISANS measurements*: The ToF‐NR characterizations were performed at the instrument D17 at the Institut Laue‐Langevin (ILL) in Grenoble, France. The GISANS studies were carried out using instrument D22 at ILL. The sample environment used for the SR measurement was also used for both neutron‐based characterizations to keep the experimental conditions consistent.^[^ ^54^ ^]^ More details about the measurement settings and the subsequent data analysis can be found in the Supporting Information. The SLD of PNIPAM was calculated and found to be 0.81 × 10^−6^ Å^−2^. The SLD of Pt‐CN was estimated from the elemental constitution of Pt‐CN measured by energy‐dispersive X‐ray analysis (EDX) (given in Table S1, Supporting Information) and found to be 7.244 × 10^−6^ Å^−2^. The SLD contributions of the substrate were maintained at constant values of 2.065 × 10^−6^ Å^−2^ for the Si substrate and 3.25 × 10^−6^ Å^−2^ for the SiO_2_ native oxide layer on the surface of the substrate.^[^ ^56^ ^]^ Regarding the SLD of the solvents involved during the experiments, 6.335 × 10^−6^ Å^−2^ and −0.561 × 10^−6^ Å^−2^ were calculated as SLD for D_2_O and H_2_O, respectively.^[^ ^60^ ^]^

*Photocatalytic water splitting and H2 production via GC*: The water‐splitting reaction was conducted in a custom‐made reactor cell connected to a gas chromatograph, allowing a high relative humidity in the cell and light irradiation. The complete setup is described in the Supporting Information. The temperature of the reactor cell was controlled using a circulating thermal bath and maintained at 14 °C throughout the experiments. The channel of the cell was filled with DI H_2_O, enabling the natural diffusion of water vapors, resulting in a high relative humidity in the sample environment. Once closed, the airtight cell was filled with argon (Ar) gas, the selected carrier gas in operation with the GC. The water vapor sorption by the hybrid hydrogels was set to 24 h, which was followed by 4 h of light irradiation. The light exposure was supplied by AM 1.5 sunlight at 100 mW cm^−2^ irradiance (Cermax Xenon lamp, Excelitas Technologies, Pittsburg, U. S. A.), placed 20 cm away from the sample environment described in the Supporting Information. After the light irradiation, 5 cm^3^ from the headspace of the cell was injected into the gas chromatograph to measure the amount of H_2_ produced.

## Supporting Information

The supporting Information contains the following: Pt doped carbon nitride synthesis and characterizations, sample preparation steps, SR measurement details, sample environment details utilized for SR, ToF‐NR, GISANS, and GC; SR, ToF‐NR, GISANS, and photocatalytic water splitting measurement details; thin film model used for ToF‐NR data fits;GISANS analysis and fitting procedure; structures ‘abundance; mass estimation of Pt‐CN in the thin‐film samples.

## Conflict of Interest

The authors declare no conflict of interest.

## Author Contributions

**Morgan P. Le Dû**: writing—original draft, conceptualization, formal analysis, investigation, and validation. **David P. Kosbahn**, **Thomas Baier**, **Julija Reitenbach**, **Qi Zhong**, and **Apostolos Vagias**: measurements (contributions to the ToF‐NR and GISANS). **Robert Cubitt**: resources (beamtime for Tof‐NR and GISANS). **Narendra Chaulagain** and **Karthik Shankar**: material (synthesis of the Pt‐doped gCN). **Hagen Übele**: measurement (GC). **Katharina Krischer**: resources (GC). **Peter Müller‐Buschbaum**: conceptualization, writing—review and editing, supervision, resources, project administration, and funding acquisition.

## Supporting information

Supplementary Material

## Data Availability

The data that support the findings of this study are available from the corresponding author upon reasonable request.
