# Supplementary material for: Platinum‐Doped Carbon Nitride‐Loaded Poly(N‐Isopropylacrylamide) Hydrogel Thin Films for Green Hydrogen Production Systems: Morphological Study for Higher Efficiency
Source: ChemSusChem. 2025 Sep 1;18(20):e202501550. doi: 10.1002/cssc.202501550 (PMC12548937; doi:10.1002/cssc.202501550)
Supplement: Supplementary file 1 — Supplementary Material [file CSSC-18-e202501550-s001.pdf]

Supporting Information

Platinum Doped Carbon Nitride loaded Poly(*N*-isopropylacrylamide) Hydrogel Thin Films for Green Hydrogen Production Systems: Morphological Study for higher Efficiency

Morgan P. Le Dû[1], David P. Kosbah[1], Thomas Baier[1], Julija Reitenbach[1], Qi Zhong[2], Apostolos Vagia[3], Robert Cubitt[3], Narendra Chaulagain[4], Karthik Shankar[4], Hagen Übele[5], Katharina Krischer[5], Peter Müller-Buschbaum[1]*

[1]Technical University of Munich, TUM School of Natural Sciences, Department of Physics, Chair for Functional Materials, James-Franck-Str. 1, 85748 Garching, Germany

[2] National Base for International Science and Technology Cooperation in Textiles and Consumer-Goods Chemistry & Zhejiang Provincial Engineering Research Center for Green and Low-carbon Dyeing & Finishing, Zhejiang Sci-Tech University, 310018 Hangzhou

[3] Institut Laue-Langevin, 71 avenue des Martyrs, 38042 Grenoble, France

[4]Department of Electrical and Computer Engineering, University of Alberta, Edmonton, AB T6G 1H9, Canada

[5]Technical University of Munich, TUM School of Natural Sciences, Department of Physics, Nonequilibrium Chemical Physics, James-Franck-Str. 1, D-85748 Garching, Germany

*Corresponding author: muellerb@ph.tum.de

Pt Doped Carbon Nitride Synthesis and Characterizations.

The platinum (Pt)-doped carbon nitride (CN) was synthesized using a modified approach from a previous report, as depicted in Figure S1. Specifically, 1.8 g of urea (99%, Fischer Scientific Acros., Germany) was ground into a fine powder with 0.79 g of citric acid (99%, Fischer Scientific Acros., Germany) and 50 mg of platinum acetylacetonate (Pt $(acac)_2$) (98%, Fischer Scientific Acros., Germany) in a mortar and pestle for 30 minutes. The resulting fine powder mixture was then placed in an acid digestion vessel and heated inside the oven at a temperature of 180 °C. The mixture was held at this temperature for 2 hours. After the reaction, the sample was allowed to cool back to room temperature. The final product was a dark green powder, which was then cleaned multiple times with methanol and subsequently dried overnight in a vacuum oven.

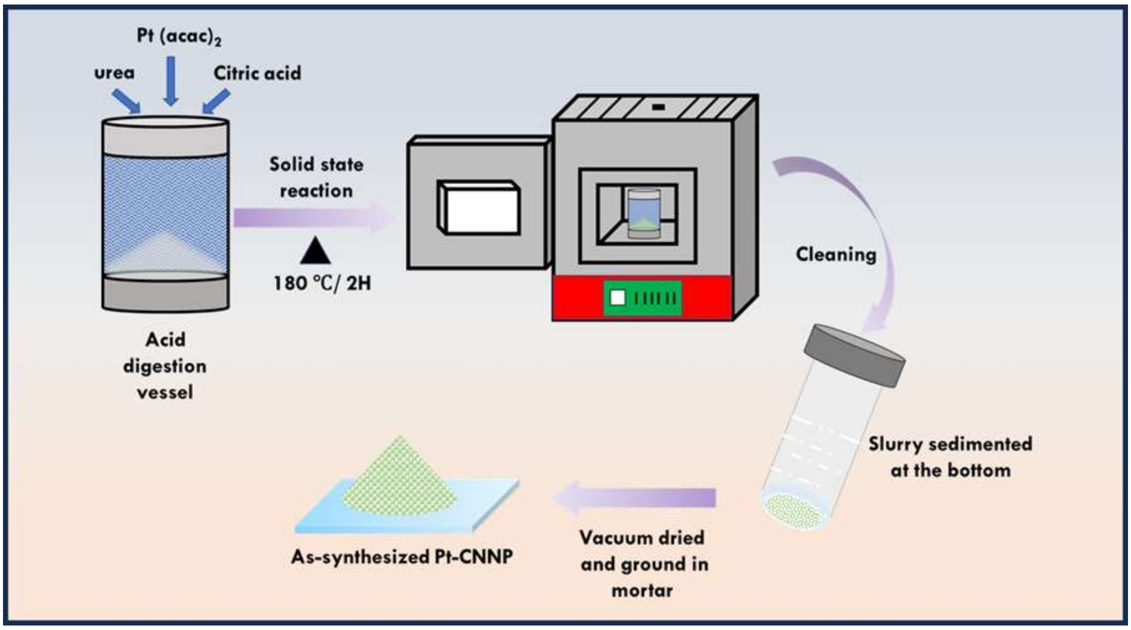

Figure S1. Schematic diagram representation of Pt-doped carbon nitride synthesis.

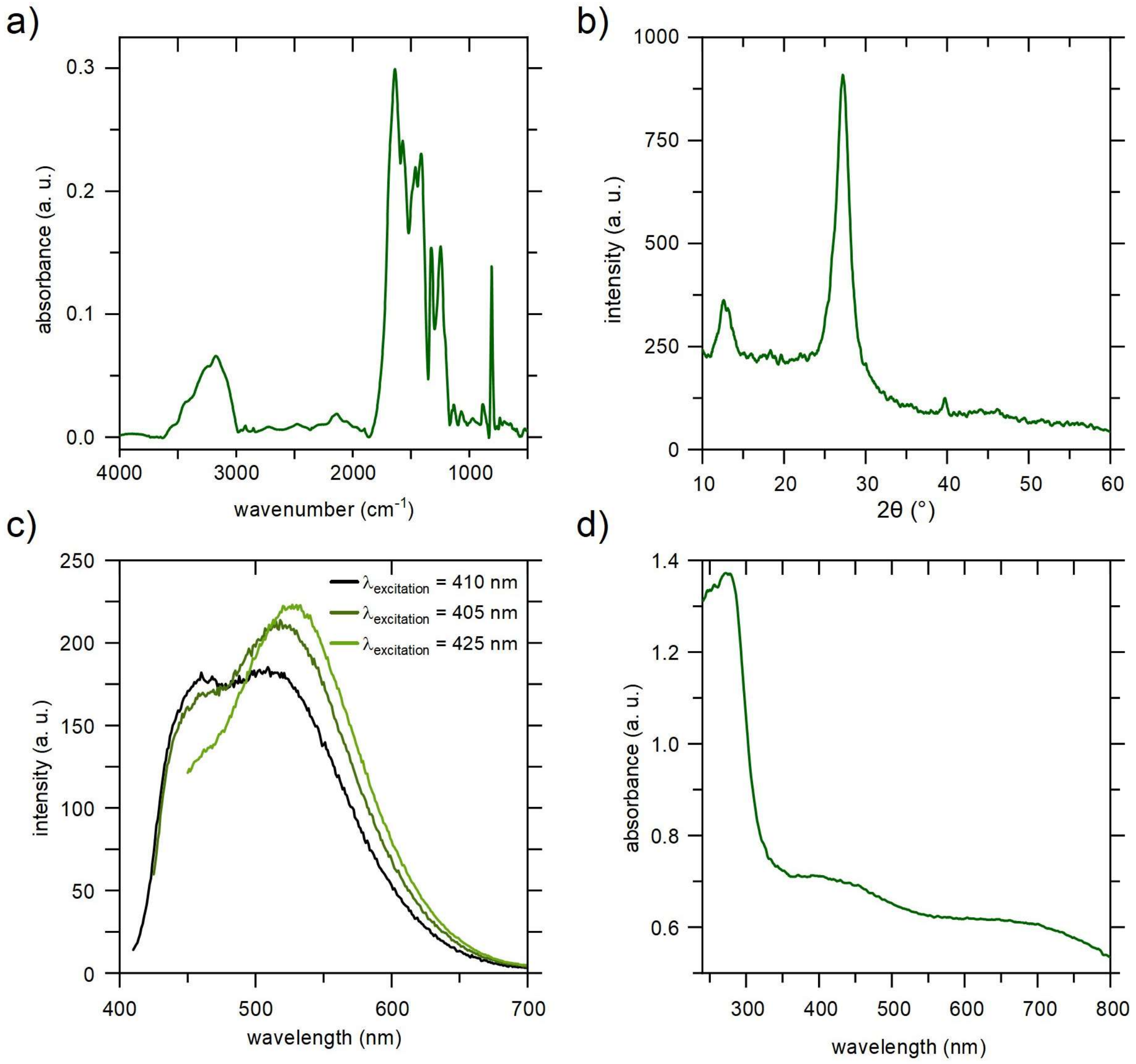

Figure S2. Characterization of the Pt-doped carbon nitride. (a) Fourier-transform infrared (FT-IR) spectrum, (b) X-ray diffractogram, (c) photoluminescence (PL) spectrum, and (d) diffuse reflectance absorption (DRS) spectrum.

Figure S2a shows absorption bands at 1570 and 1640 cm^{-1}, which are attributed to C=N stretching, while the two bands at 1327 and 1460 cm^{-1} to aromatic C-N stretching. The 1230 and 1415 cm^{-1}

peaks are attributed to –CO stretching and –OH bending mode, respectively, and originate from the carboxylic acid end groups. The peak at 809 cm^{-1} is assigned to the triazine ring mode, corresponding to condensed CN heterocycles. A broad band near 3000 ~ 3500 cm^{-1} corresponds to the stretching modes of $–NH_2$ and –OH. Figure S2b depicting the X-ray diffractogram shows a peak at 27.3 ° reported as the (002) plane, equivalent to a d-spacing of 0.327 nm, which is commonly attributed to the carbon nitride sheet spacing. The peak observed at 12.8 ° is ascribed to the (100) plane, where the d-spacing is 0.692 nm and is associated with the intralayer spacing. The PL spectrum observed in Figure S2c shows 2 emissions near 450 nm and 500 nm under an excitation wavelength of 410 nm, suggesting two emission pathways. The 450 nm emission peak is reported to the conduction-valence band recombination, while the 500 nm peak is attributed to n–π* transition related to the N lone pair electrons in the polymeric carbon nitride structure. The Ultraviolet – visible (UV-vis) absorption measured by diffuse reflectance spectroscopy (DRS) depicted in Figure S2d shows a strong absorption peak in 250 and 330 nm, which has been assigned to the π–π* electronic transition of the sp^2 hybridized carbons of the C=N and C=C bonds within the tris-*s*-triazine unit. Another absorption peak is found between 370 and 480 nm and is ascribed to n–π* electronic transition of the aromatic nonbonding orbitals for the C=N and C=O bonds. The last shoulder is observed between 600 and 800 nm in literature in metal-doped CN and is referred to as a metal-to-ligand charge transfer process that broadens the light absorption close to the near infrared region.[1] In addition, Figure S3 shows the Raman spectrum of the Pt-doped CN, where the D band is observable at 1310 cm^{-1}, which is a characteristic Raman mode of all graphitic systems; it originates from defects and disorder in the carbon lattice and the double resonance processes around the K point of the Brillouin Zone. The Raman spectrum also exhibits the G bands at 1585 cm^{-1}, a mode corresponding to the Raman E_{2g} optical phonon. Generally, the intensity ratio (I_D/I_G) indicates the degree of disorder in the aromatic structure of graphitic materials. A high I_D/I_G

ratio represents a high structural regularity. In the present case, I_D/I_G = 0.7 (<1), indicating an inherent disorder of the catalyst material. Such a low intensity ratio is expected as CN material has been functionalized with -COOH groups and doped with Pt.

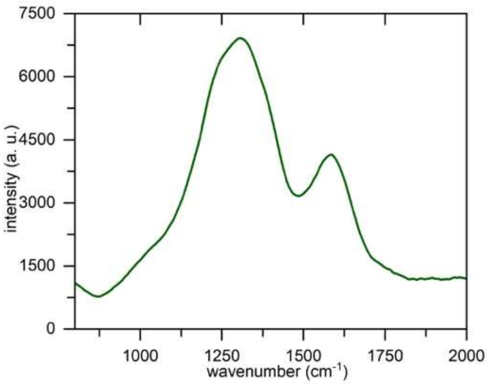

Figure S3. Raman spectrum of Pt-doped CN.

Table S1. Weight and associated atomic percentage measured by energy-dispersive X-ray analysis.

Element	*Weight percentage (Wt%)*	*Atomic percentage (At%)*
Carbon (C)	51.3	57.3
Oxygen (O)	26.8	22.5
Nitrogen (N)	21.0	20.1
Platinum (Pt)	0.7	0.05
Contaminant: Potassium (K)	0.3	0.05

The elemental composition of Pt-doped CN is estimated from energy dispersive X-ray analysis (EDX), and the resulting elemental weight percentages are found in Table S1. The amount of

oxygen and the high percentage observed for carbon can be explained by the -COOH functional end groups of the synthesized catalyst.

Sample Preparation Steps

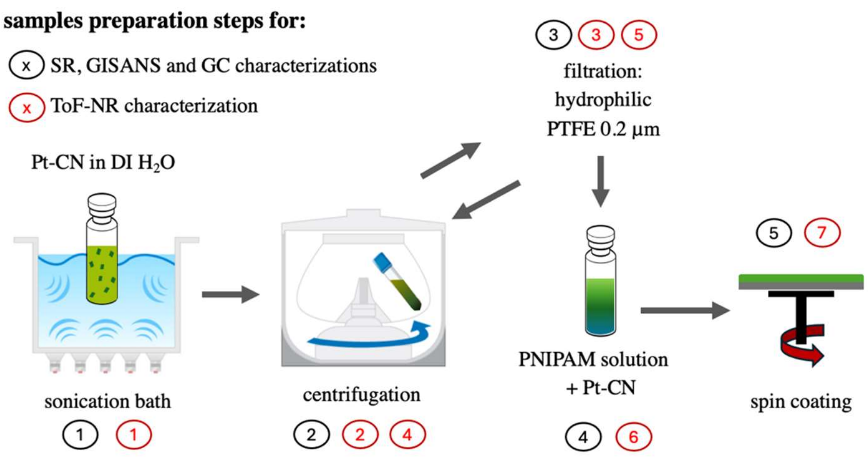

Figure S4. Detailed sample preparation steps for the different characterization techniques used in the study.

The spectral reflectance (SR), grazing incidence small-angle neutron scattering (GISANS) and gas chromatography (GC) characterizations are performed on the catalyst-loaded samples prepared with the following steps: (1) sonication of carbon nitride aqueous solution (2) centrifugation of the carbon nitride solution, (3) filtration of the supernatant phase of the carbon nitride solution using a hydrophilic polytetrafluoroethylene (PTFE) membrane, (4) introduction of the carbon nitride solution with a poly(*N*-isopropylacrylamide) (PNIPAM) solution, and (5) film deposition of the resulting mixture by spin coating.

The time-of-flight neutron reflectometry (ToF-NR) characterization is performed a catalyst-loaded sample prepared with the following steps: (1) sonication of carbon nitride aqueous solution (2) centrifugation of the carbon nitride solution, (3) filtration of the supernatant phase of the carbon nitride solution using a hydrophilic PTFE membrane, (4) centrifugation of the carbon nitride solution, (5) filtration of the supernatant phase of the carbon nitride solution using a hydrophilic PTFE membrane, (6) introduction of the carbon nitride solution with a PNIPAM solution, and (7) film deposition of the resulting mixture by spin coating. As described in Figure S4, an additional round of centrifugation and filtration steps is performed for the ToF-NR sample. Such steps are necessary to ensure a high smoothness of the sample surface, which is crucial for obtaining well-pronounced Kiessig fringes in the NR, allowing the fine analysis of the vertical film distribution.

Sample Environment Utilized for Spectral Reflectance (SR) and Time-of-Flight Neutron Reflectometry (ToF-NR) and Grazing Incidence Small Angle Neutron Scattering (GISANS)

The experimental setup for SR and ToF-NR measurements involves a three-dimensional printed measurement chamber fabricated from AlMgSi alloy. The environment is equipped with aluminum windows and features liquid channels embedded within the walls of the chamber, ensuring a uniform temperature distribution around the sample location.[2],[3] A custom-designed vapor generation system connected to the sample environment facilitates a vapor-saturated gas flow with adjustable composition. This system allows the combination of dry N_2 and saturated H_2O/D_2O vapor streams to be delivered to the sample with a 1 $L\cdot min^{-1}$ flow rate. The whole setup consists of the vapor generation system and the chamber, both temperature controlled by a thermal bath cycle using the JULABO FP50 HL system (JULABO Labortechnik GmbH, Seelbach, Germany). Thereby ensuring a constant temperature of 19 °C during all *in situ* measurements. The temperature

and the relative humidity inside the sample environment were monitored with a SHT31 sensor (Sensirion AG, Stäfa, Switzerland).

Sample Environment Utilized for Photocatalytic water-splitting and gas chromatograph (GC) Description

The sample environment presented in Figure S4 is used for determining the H2 evolution rate of the studied samples. It is composed of an aluminum base elevating the whole sample environment, enabling a light irradiation from the bottom, provided by a light source 1.5G illumination (class ABA, LOT-Quantum design GmbH, 100 $W \cdot m^{-2}$). The base is mounted with a silver-coated mirror (Thorlabs, Inc., Newton, U.S.A.), enabling a high reflectance of visible light. The sample is placed between two calcium fluoride (CaF_2) windows (Edmund Optics Ltd., Nether Poppleton, York, U.K.), allowing the transmission of ultraviolet and visible light. Both the lid and the chamber are designed so that the headspace of the sample environment is reduced to a minimum when fully assembled. The chamber part (in red in Figure S4) contains a channel that is filled with water in order to build a high-humidity environment. The whole assembly is temperature-controlled by a circulating water bath monitored by a temperature sensor (Sensirion AG, Stäfa, Switzerland). The recorded temperatures during the water-splitting experiments are shown in Figure S6. The sample environment is filled with argon (Ar 5.0), and a constant overpressure of approx. 200 mbar(g) with an Alicat MC-1SCCM-D flowmeter. This device additionally monitors the loss rate of the chamber. The chamber is connected to a Trace 1310 gas chromatograph with an auxiliary oven by thermo scientific through a bespoke dynamic controlled pressure injector (dynamic CPI) constructed by S+H Analytik GmbH. This CPI consists of valve flow- and pressure-meters and a vacuum pump and allows for injecting a precisely measured sample at a set injection pressure. The dynamic CPI

generates a vacuum inside the GC columns before opening the inlet valve and measuring the amount of sample volume that flows into the columns before injection into the measuring columns. The GC contains two Thermal Conductivity Detectors (TCD) and two Flame Ionization Detectors (FID), with Hydrogen detection being performed in the second TCD (SN721450010).

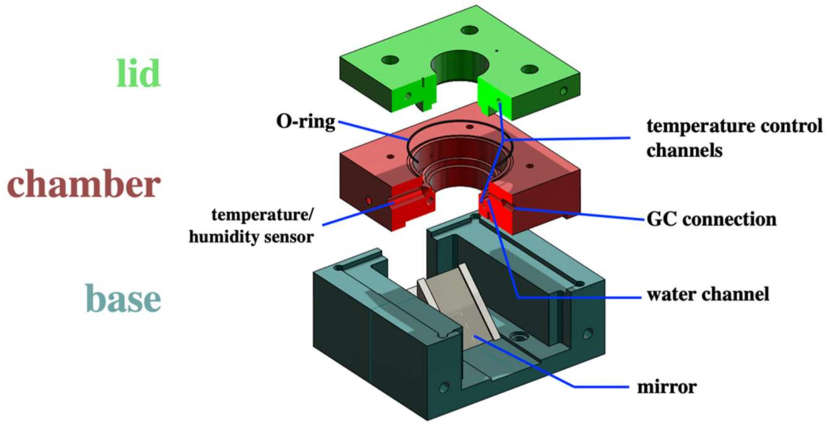

Figure S5. Three-dimensional view of the setup used for the photocatalytic water splitting experiments. The lid and the chamber are truncated for clarity.

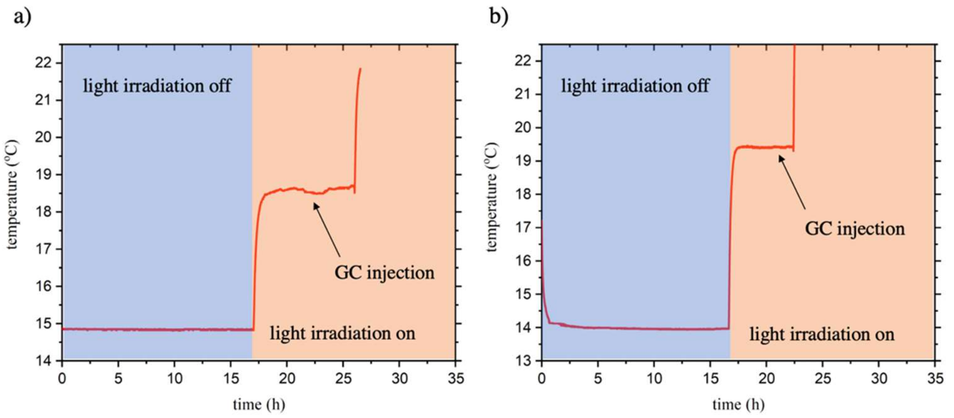

Figure S6. Temperature evolution over time during the water-splitting experiments of a) L-PtCN/PNIPAM and b) H-PtCN/PNIPAM samples. The blue areas represent the time when the AM 1.5 light irradiation was off, and the orange areas represent when the AM 1.5 light irradiation was on. The arrow indicates when the gas from the sample environment was injected into the GC.

Figure S6 shows the evolution of the temperature during the water-splitting experiments. The sharp temperature rise observed at 16.5 h originates from the strong influence of the AM 1.5 light irradiation. The light used for these experiments is AM 1.5 sunlight at 100 mW cm^{-2} irradiance, simulating the sun spectrum at sea level. Such a powerful light source induced heat in the sample environment. The temperature is kept relatively low before the light irradiation to anticipate the rise once the light is on. Therefore, once under irradiation, the sample environment is maintained well below the temperature transition of the polymer matrix (c.a. 32 ºC).[4]

SR Measurement Details

Filmetrics F20 Thin-Film Analyzer (KLA, Milpitas, U.S.A.) with a halogen lamp light source (λ = 380 – 1100 nm) was used, which consisted of an optical fiber that led the white light to a lens

assembly mounted on the top of the sample environment. The light was focused to a spot of ca. 2 mm on the sample surface. A spectrum was recorded every 10 s and was analyzed using the robust thickness fit algorithm included in the FILMeasure software (KLA Corporation, Milpitas, U.S.A.). The samples were placed for 1 h under a N_2 stream to dry the film. Then, the thickness evolution of the thin film sample was measured within the high relative humidity built in the chamber.

ToF-NR Measurement Details

For the operation of the neutron reflectometer (D17, Institut Laue-Langevin (ILL), Grenoble, France) in time-of-flight (ToF) mode, a wavelength band ranging from $\lambda = 2 – 27$ Å with a spectral resolution of $\Delta\lambda/\lambda = 10\%$ was chosen. An out-of-reflection-plane divergent incident beam was used to ensure optimal utilization of the available neutron flux.[5] Static measurements were carried out using two incident angles, namely $\alpha_i = 0.5°$ (10 min counting time) and $\alpha_i = 2.5°$ (30 min), providing coverage of a broad range of momentum transfers q_z. The sample-detector distance remained constant at the maximum value of 3.1 m for all measurements. ToF neutron reflectometry (NR) data underwent reduction using a re-binning algorithm designed for out-of-reflection-plane divergent beam experiments, thereby enhancing the resolution of the data.[6],[7]

Thin Film Model Used for ToF-NR Data Fits

The fit procedure of the ToF-NR data required an appropriate model to recreate the sample structure. A generic layer model was used, as illustrated in Figure S5.

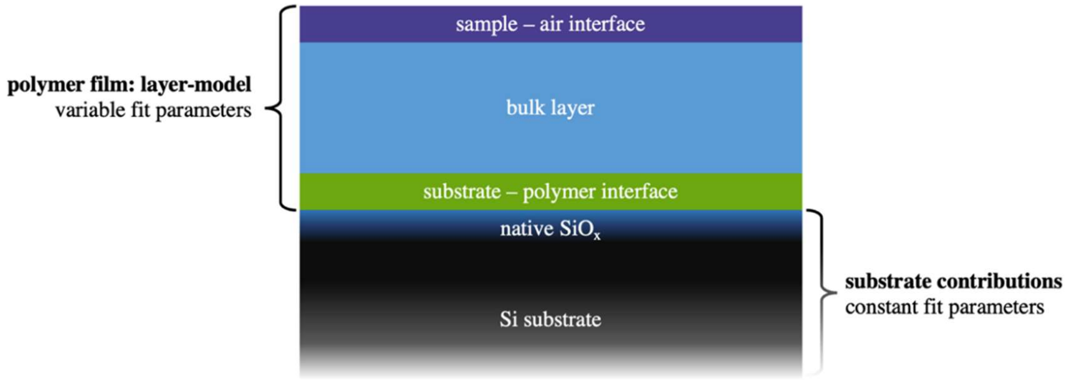

Figure S7. Layer model used to fit the ToF-NR data.

The substrate contributions, consisting of the silicon substrate and its native SiOx layer, remain the same for both samples, PNIPAM and Pt-CN/PNIPAM. Therefore, the fit parameters are kept constant.

GISANS Measurement Details

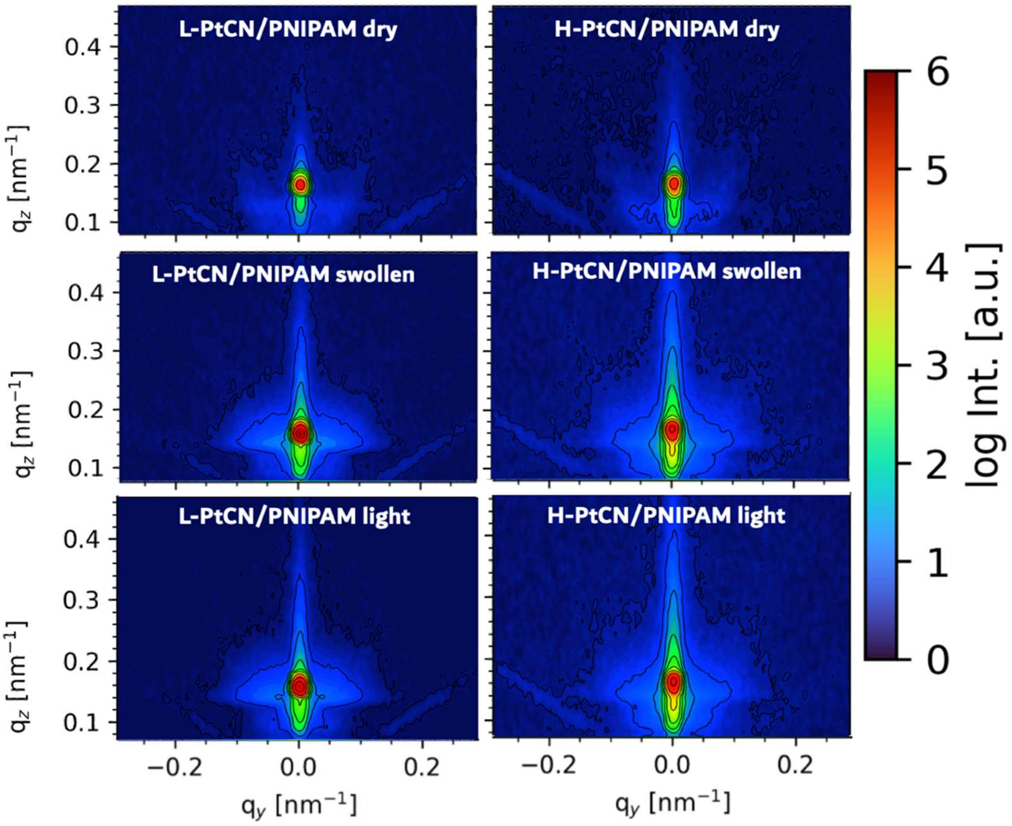

Figure S8. 2D GISANS data recorded for L-PtCN/PNIPAM (left column) and H-PtCN/PNIPAM (right column) samples at each stage of the characterization (dry, swollen, and illuminated).

The GISANS characterization was performed at the instrument D22 (Institut Laue-Langevin (ILL), Grenoble, France) with a wavelength of λ = 6 Å and a spectral resolution of $\Delta\lambda/\lambda$ = 10%. The sample detector distance was set to SDD = 17.6 m and the collimation length to CL = 20 m. The dimensions of the primary beam were 0.1 mm (along q_z direction) × 10 mm (along q_y direction) to obtain the highest neutron flux delivered to the sample. The incident angle of the neutron beam was α_i = 0.45°. For the so-called "static" measurement, operated at the dry, hydrated, and light-irradiated state of the samples, the sample-beam exposure time was set to 60 minutes with 6

repetitions. The resulting static measurements are shown in Figure S6. While the so-called "kinetic" measurements, which were operated during the D_2O vapor exposure and the UV-light irradiation, were set to a neutron beam exposure time of 10 minutes with 12 and 10 repetitions for the hydration and the UV-light irradiation, respectively.

The 6 static measurements performed on H-PtCN/PNIPAM and L-PtCN/PNIPAM films during the UV-light irradiation are individually considered to further verify the stability of the samples. Hence, horizontal line cuts are performed at the Yoneda region of each 2D GISANS data to obtain the associated reduced 1D intensity profiles. The resulting profiles are shown in Figure S9.

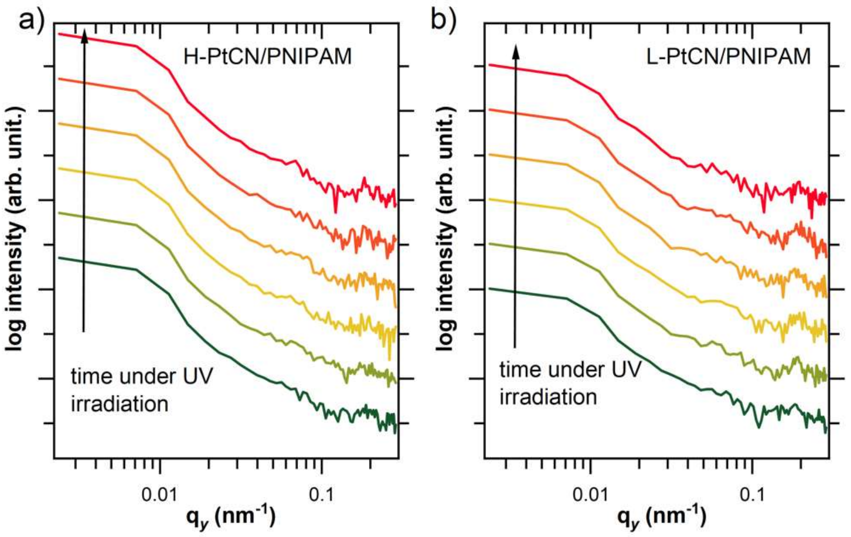

Figure S9. Horizontal line cuts of the 2D GISANS data at the Yoneda position of a) H-PtCN/PNIPAM and b) L-PtCN/PNIPAM films for the UV-light irradiation. The curves are shifted along the y-axis for clarity of the presentation.

The 1D intensity profiles along q_y shown in Figure S9 do not present apparent differences, suggesting that the samples are not experiencing degradation during the considered 6 h of UV-light irradiation.

GISANS Analysis and Fit Procedure

The 2D GISANS data was reduced to 1D intensity profiles horizontally, by proceeding to a line cut along q_z at the Yoneda region (at the critical angle of total reflection), and vertically with a line cut at $q_y = 0$. The commonly referred Yoneda region/peak is observed close to the critical angle of the probed components, which is therefore scattering length density (SLD) dependent. For a multi-component system with inhomogeneous dispersion, individual Yoneda peaks can be distinguished (depending on the scattering contrast of the considered components). The vertical line cut typically extracts the SLD distribution in the direction normal to the substrate and enables the location of the Yoneda peak(s). In contrast, the horizontal line cut provides specific information about the lateral morphology from I(q_y). The areas of interest in the 2D GISANS data are depicted in Figure S7.

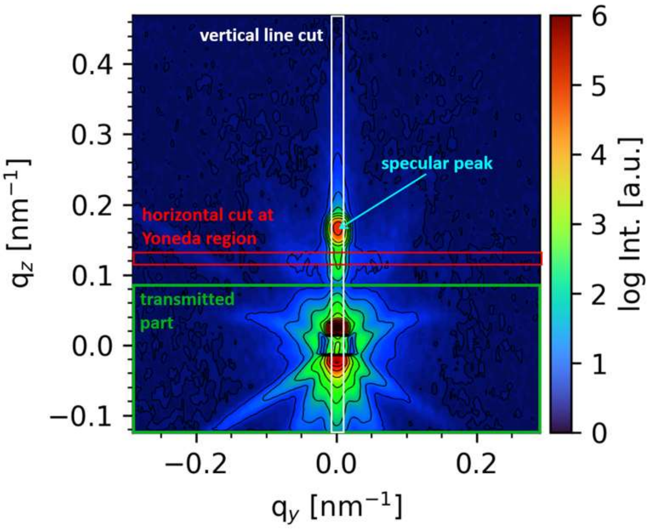

Figure S10. Exemplary 2D GISANS data. The green box shows the transmitted part of the neutron beam. The white box shows the vertical line cut at $q_y = 0$ nm$^{-1,}$ where the intensity is probed along q_z. The red box shows the Yoneda region where the horizontal line cuts to obtain the intensity along q_y.

The domains are modelled with cylinders with radius R, and their size distribution, as well as their corresponding mean center-to-center distances D, are derived from the I(q_y) profile of the integrated horizontal line cut. A model is used to extract R and D and follows the expression:

$$\mathrm{I}\,(\mathrm{q}) \propto \sum_{\mathrm{i}} \mathrm{N_i} \langle |\mathrm{F}\,(\mathrm{q}, \mathrm{R_i})|^2 \rangle\, \mathrm{S}\,(\mathrm{q}, \mathrm{D_i})$$

where F (q, R_i) and S (q, D_i) are the form factor and structure factor, respectively. Due to a multi-layered/component system, multiple scattering and reflection events occur for an individual neutron; hence, the so-called "distorted-wave Born approximation" (DWBA) is used. The DWBA accounts for possible combinations of scattering and reflection events for an incoming and outgoing probe of the neutron beam. The domains are assumed to be monodisperse within the beam coherence length and have an average form factor following a Gaussian standard deviation σ_i:

$$\langle |F(q,R_i)|^2 \rangle = \frac{\int \sigma_i(R)|F(q,R)|^2 dR}{\int \sigma_i(R)\, dR}$$

In the present study, a cylindrical form factor is expected due to the sheet-like shape of the catalytic centers

$$|F_{cyl}(q_y)|^2 = \left(R\,\frac{J_1(q_y, R)}{q_y}\right)^2$$

with J_1, the Bessel function of the first kind. To further model the intensity profile of the horizontal line cuts and extract the center-to-center domain distances within a 1D paracrystal lattice (including cumulative disorder to induce a loss of long-range order), the Hosemann interference function is applied

$$S(q_y) = \frac{1 - \exp(\pi\sigma_D^2 D^2 q_y^2)^2}{1 + \exp(\pi\sigma_D^2 D^2 q_y^2)^2 - 2\exp(\pi\sigma_D^2 D^2 q_y^2)\cos(q_y D)}$$

where σ expresses the standard deviation from a mean center-to-center distance D and is defined by a Gaussian distribution. The presented model consists of a sum of three distinct domains. The model construction and the corresponding fit procedure of the recorded scattering data are performed via a customized Python-based script.

Structure Abundance

The overall contribution of the scatterers within the films is described in Figure S8, showing the domain size distribution for both samples in the probed states.

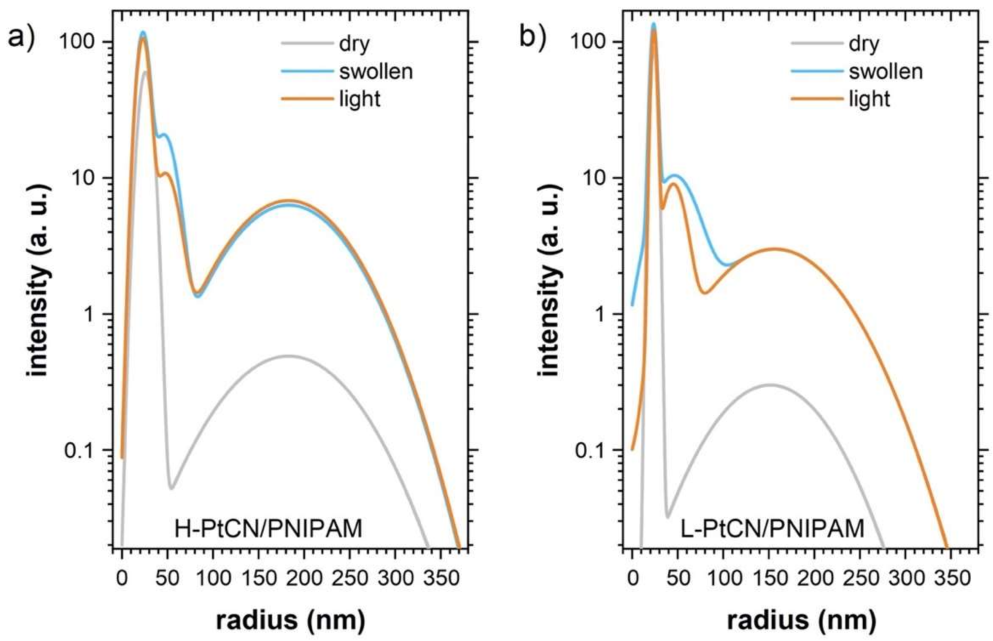

Figure S11. Domain size distributions for (a) L-PtCN/PNIPAM and (b) H-PtCN/PNIPAM film in a dry (gray), swollen (blue), and illuminated (yellow) state.

Mass Estimation of Pt-CN in the Thin-Film Samples

The H_2 evolution rate is commonly defined by the amount of produced H_2 given in micromoles (µmol) per gram of catalyst present in the samples per hour of light irradiation (µmol·g^{-1}·h^{-1}). Considering the filtration and the centrifugation steps operated on the Pt-CN solution before its introduction into the polymer solution, plus the spin coating deposition techniques, which lead to some material losses, the final mass present in the samples needs to be carefully estimated to be able to translate the obtained H_2 generation rate. For this purpose, the physical dimensions of the films need to be taken into account: we define $z_{\text{H-PtCN}}$ and $z_{\text{L-PtCN}}$, the thickness measured by SR for H-PtCN/PNIPAM and L-PtCN/PNIPAM samples. Both samples are coated on a square glass substrate of length L_{film}. Hence, the area of the samples and their volume can be found as A_{film} =

L_{film}^2 and $V_{film\ H/L\text{-}PtCN} = z_{H\text{-}PtCN}$ (or $z_{L\text{-}PtCN}$) × A_{film}. Then, the concentration of the used material in the sample is given as C_{PNIPAM} and $C_{Pt\text{-}CN}$ for PINPAM and catalyst concentration, respectively. $C_{Pt\text{-}CN}$ is the catalyst concentration obtained after the filtration and centrifugation steps and is determined from a calibration curve built with ultra-violet visible-light spectroscopy using several solutions of known concentration, as shown in Figure S9.

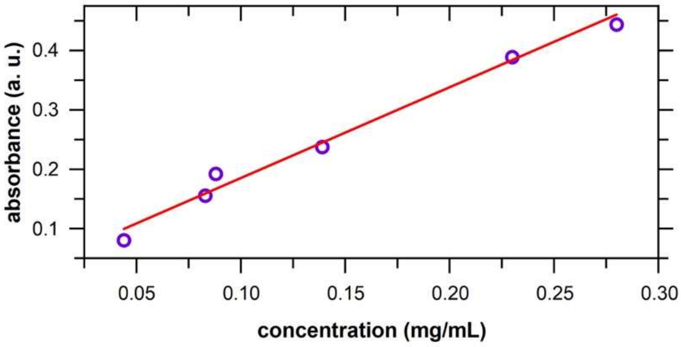

Figure S12. Absorbance for Pt-CN solution as a function of its concentration in aqueous solution. The circles represent the recorded data, and the solid red line is the associated linear fit.

Both H-PtCN/PNIPAM and L-PtCN/PNIPAM samples contain different amounts of catalyst, so $C_{H\text{-}PtCN}$ and $C_{L\text{-}PtCN}$ are introduced as $C_{H/L\text{-}PtCN} = C_{Pt\text{-}CN} \times V_{H/L\text{-}PtCN} / V_{solution}$, where $V_{H/L\text{-}PtCN}$ is the volume of Pt-CN solution introduced in the final solution of volume $V_{solution}$.

The volume ratio of the catalyst in the solutions used for the films' deposition is defined as:

$$R_{H/L-PtCN} = \frac{C_{H/L-PtC} / d_{Pt-C}}{\left(C_{H/L-PtC} / d_{Pt-C}\right) + \left(C_{PNIPAM} / d_{PNIPAM}\right)},$$

where $d_{Pt\text{-}CN}$ and d_{PNIPAM} are the mass density of the catalyst and PNIPAM, respectively.

The volume occupied by the catalyst in the H-PtCN/PNIPAM and L-PtCN/PNIPAM samples after the spin coating deposition is now expressed as $V^{*}_{H/L\text{-}PtCN} = R_{H/L\text{-}PtCN} \times V_{film\ H/L\text{-}PtCN}$. Finally, the mass of the Pt-CN catalyst in the samples reads $m_{H/L\text{-}PtCN} = V^{*}_{H/L\text{-}PtCN} \times d_{Pt\text{-}CN}$. Table S2. summarized the values used for the calculation and the obtained masses of the catalyst present in the samples.

Table S2. Parameters used to estimate the mass of the catalyst in the samples and the obtained catalyst masses.

$z_{\mathbf{H\text{-}PtCN/PNIPAM}}$	245.3 ± 0.1 nm
$z_{\mathbf{L\text{-}PtCN/PNIPAM}}$	239.7 ± 0.1 nm
$L_{\mathbf{film}}$	3.0 ± 0.1 cm
$C_{\mathbf{Pt\text{-}CN}}$	4.30 ± 0.02 mg·mL^{-1}
$C_{\mathbf{PNIPAM}}$	36.3 ± 0.1 mg·mL^{-1}
$V_{\mathbf{H\text{-}PtCN}}$	0.300 mL
$V_{\mathbf{L\text{-}PtCN}}$	0.150 mL
$V_{\mathbf{solution}}$	3.3 mL
$d_{\mathbf{PNIPAM}}$	1.1 g·cm^{-3}
$d_{\mathbf{Pt\text{-}CN}}$[8]	2.3 g·cm^{-3}
$m_{\mathbf{H\text{-}PtCN}}$	2.6 ± 0.2 μg
$m_{\mathbf{L\text{-}PtCN}}$	1.27 ± 0.09 μg

REFERENCES

[1] Li, C., Wang, Y., Li, C., Xu, S., Hou, X., Wu, P., *ACS applied materials & interfaces* **2019,** *11* (23), 20770-20777.

[2] Widmann, T., Kreuzer, L. P., Mangiapia, G., Haese, M., Frielinghaus, H., Müller-Buschbaum, P., *Review of scientific instruments* **2020,** *91* (11).

[3] Widmann, T., Kreuzer, L. P., Kühnhammer, M., Schmid, A. J., Wiehemeier, L., Jaksch, S., Frielinghaus, H., Löhmann, O., Schneider, H., Hiess, A., Klitzing, R. v., Hellweg, T., Müller-Buschbaum, P., *Applied Sciences* **2021,** *11* (9), 4036.

[4] Le Dû, M. P., Reitenbach, J., Kosbahn, D. P., Spanier, L. V., Cubitt, R., Henschel, C., Laschewsky, A., Papadakis, C. M., Müller-Buschbaum, P., *Macromolecules* **2025,** *58* (2), 1000-1010. DOI 10.1021/acs.macromol.4c02802.

[5] Cubitt, R., Fragneto, G., *Applied Physics A* **2002,** *74* (1), s329-s331. DOI 10.1007/s003390201611.

[6] Cubitt, R., Saerbeck, T., Campbell, R. A., Barker, R., Gutfreund, P., *Journal of Applied Crystallography* **2015,** *48* (6), 2006-2011.

[7] Gutfreund, P., Saerbeck, T., Gonzalez, M. A., Pellegrini, E., Laver, M., Dewhurst, C., Cubitt, R., *Journal of Applied Crystallography* **2018,** *51* (3), 606-615.

[8] Jiang, X., Zhuang, C., Zhao, J., Jiang, X., *Diamond and Related Materials* **2012,** *23*, 44-49. DOI https://doi.org/10.1016/j.diamond.2011.12.014.
